# Pyrvinium pamoate can overcome artemisinin’s resistance in anaplastic thyroid cancer

**DOI:** 10.1186/s12906-021-03332-z

**Published:** 2021-05-28

**Authors:** Yitian Li

**Affiliations:** Research Department of Jining Medical University, He Hua Road 133, Jining, Shandong China

**Keywords:** Anaplastic thyroid cancer, Artemisinin, Pyrvinium pamoate, Apoptosis, Drug resistance

## Abstract

**Background:**

Anaplastic thyroid carcinoma is a highly lethal subtype of thyroid cancer without effective therapies. Drug resistance in anaplastic thyroid carcinoma poses a significant problem. Although artemisinin exerts antitumor effects, but its efficacy in anaplastic thyroid carcinoma is unknown.

**Methods:**

We used RNA sequencing to identify differentially expressed genes. Next, we determined the cause of ART resistance by testing the expression and activity of β-catenin, and enhanced ART activity with a WNT signaling inhibitor.

**Results:**

Artemisinin suppressed the growth of BHT-101 but not human thyroid anaplastic carcinoma (CAL-62) cells. The mechanism of artemisinin resistance in CAL-62 was associated with the aberrant activation of WNT signaling. Pyrvinium pamoate, an inhibitor of WNT signaling, was used to overcome ART resistance in CAL-62 cells. The combination of artemisinin and pyrvinium pamoate suppressed the growth of CAL-62 cells and induced the apoptosis.

**Conclusions:**

Our study is the first to prove the efficacy of ART as monotherapy or in combination with PP in the management of anaplastic thyroid cancer, and that the inhibition of WNT signaling may overcome ART resistance.

**Supplementary Information:**

The online version contains supplementary material available at 10.1186/s12906-021-03332-z.

## Background

Thyroid cancer (TC) is one of the most common endocrine cancers worldwide with an increasing incidence in recent years. According to the Global Cancer Statistics 2020, TC has been ranked as the fifth most common malignant tumor in women [1]. Anaplastic thyroid cancer (ATC) is a subtype of TC that accounts for only 2–3% of TC [2]; However, it is one of the most aggressive forms of TC. The median survival time of ATC is only 5 months [3]. ATC is considered invasive or metastatic at diagnosis, regardless of the tumor size, lymph node metastases, or distant metastases, and is classified as stage IV TC by the American Joint Committee on Cancer [4]. Common therapeutic strategies for the management of ATC include treatment with BRAF inhibition [5], vascular endothelial growth factor inhibitiors [6], epidermal growth factor receptor inhibitiors [7], and antitumor immunotherapy that targets PD-1 and PD-L1 [8].

However, the clinical benefits after drug threrapy are transient and not long term, and are often associated with various adverse effects. The therapeutic effects are not significant when these drugs are used as monotherapy for the treatment of ATC. As the development of synthetic drugs entails high costs and is often associated with high toxicity and side effects, it is essential to identify antitumor drugs that are efficacious, nontoxic, and inexpensive to synthesize.

Artemisinin (ART) is a phytochemical that has been recently reported to exert antitumor effects and comparatively few side effects. ART is a sesquiterpene lactone compound containing a peroxide bridge without nitrogen atoms and is effective in the treatment of malaria [9]. Several studies have been carried in the past decade to determine the antitumor mechanism of ART-like compounds; however, their precise mechanism of action is still unknown. Although current studies show that the main antitumor mechanisms of ART include response to oxidative damage, cell-cycle arrest, induction of apoptosis, inhibition of angiogenesis and metastasis, and invasion [10], its mechanism of action in ATC is still unknown. We investigated the mechanism of ART in ATC and attempted to provide new alternative drugs for the management of ATC. Moreover, due to acquired tolerance, drug resistance to ATC is a common problem. We also attempted to determine and overcome the mechanism of drug resistance.

## Methods

### Anaplastic thyroid cancer cell lines and culture

CAL-62 and BHT-101 cell lines were purchased from FuHeng Cell Center (Shanghai, China) and cultured in DMEM medium (KeyGen BioTECH, Jiangsu, China) supplemented with 10% fetal bovine serum (FBS) (Gibco, NY, USA) for CAL-62 and 20% FBS for BHT-101. A total of 4–6 × 10^4^ cells/mL cells were plated into culture dishes (Corning, NY, USA) and incubated in atmosphere of 5% CO_2_ at 37 °C for 12–24 h before the experiments were performed. For immunofluorescent labeling, several cell-bearing coverslips were prepared under similar conditions by using coverslip preparation dishes (JET Bio-Filtration, Guangzhou, China) for multiple experiments.

### Reagents and cell treatment

ART (Yuanye Bio-Technology, Shanghai, China) and pyrvinium pamoate (PP) (GlpBio, CA, USA) were dissolved in dimethylsulfoxide (DMSO; KeyGen BioTECH, Jiangsu, China) and diluted with culture medium to yield concentrations of 100 mM and 100 μM, respectively, prior to use. ATC cells were treated with 100 μM ART or 80 nM PP combined with 150 μM ART for 48 h and observed at 12-h intervals. Normally cultured cells or those treated with 0.2% DMSO were used as negative controls. Cell viability was checked at 24 h or 48 h. The cells on the cell-bearing coverslips were fixed in 4% paraformaldehyde (pH 7.4) for the immunofluorescence assay. All experimental groups were tested in triplicate, and each experiment was performed at least three times. The data were summarized and statistically analyzed.

### Evaluation of cell proliferation

To evaluate the cellular response of CAL-62 and BHT-101 to ART, CCK-8 cell proliferation assay was performed on 96-well plates bearing CAL-62 and BHT-101 cells treated with or without different concentrations (50, 100, 150, and 200 μM) of ART for 24 h or 48 h using a previously described method [11]. Flow cytometry (Beckman Coulter, CA, USA) was used to determine the cell cycle phase after treatment with 100 μM ART for 48 h, and the data were analyzed using MODFIT software (Version 5.0, Verity Software House Inc., ME, USA). Cells treated with 0.2% DMSO were used as control.

### Sample preparation for RNA-seq

CAL-62 and BHT-101 cells were treated with 100 μM ART for 48 h and observed at 12-h intervals. Normally cultured cells were used as the background and untreated control groups. Three samples were collected from each group (Control and Treatment) and sent to RNA-seq (Applied Protein Technology, Shanghai, China) over dry ice to obtain RNA-seq gene data.

### Protein preparation and Western blotting

Western blotting was performed using antibodies against Cyclin D1 (1:2000; proteintech, Illinois, USA), ANGPTL2 (1:5000; proteintech), EGR1 (1:2000; Affinity Biosciences, Ohio, USA), β-catenin (1:5000; proteintech), WNT7B (1:3000; Affinity Biosciences), FZD8 (1:1:1000; Affinity Biosciences), caspase 3 (1:2000; Affinity Biosciences), active-caspase 3 (1:2000; Affinity Biosciences), and β-actin (1:8000; Abclonal, Wuhan, China). The experiment was performed using a previously published method [12]. Briefly, protein samples obtained from the CAL-62 and BHT-101 cells after treatment with 100 μM ART or 0.2% DMSO treatment for 48 h. The cells were lysed using RIPA lysis buffer (Beyotime Biotechnology, Shanghai, China) for 15 min on ice. The nucleoprotein was obtained from CAL-62 cells after treatment with 100 μM ART for 48 h by following the manufacturer’s instructions in the cellular nucleoprotein extraction kit (Beyotime Biotech). Protease and the phosphatase inhibitor (50×, Beyotime Biotech) were added to the RIPA lysis buffer in a 1:50 ratio. PAGE Gel Fast Preparation kit (EpiZyme, Shanghai, China) was used for SDS-PAGE. Samples (16 μL) were loaded onto the gel in each lane. Protein-free Rapid Blocking buffer (EpiZyme) was used to block the membranes (30 min to 1 h). Next, the membrane was incubated with the unconjugated primary antibody overnight at 4 °C. The membrane was washed three times with 1 × TBST (Beyotime Biotech) for 5 min and incubated with the HRP-linked antibody (1:6000–10,000) for 2 h at room temperature (23–25 °C). Millipore Immobilon Western (Merck Life Science, USA) was incabated for 1 min at room temperature and images were captured.

### Immunofluoresce labeling

Immunofluorescent labeling was performed on cell-bearing coverslips using the method described previously [12]. The CAL-62 cells on cell-bearing coverslips were treated with 100 μM ART or 0.2% DMSO for 48 h. The working concentration of rabbit anti-β-catenin used for immunofluorescence staining was 1:500. Briefly, the cell-bearing coverslips were washed in phosphate-buffered saline (PBS, pH 7.4), incubated in 3% H_2_O_2_ for 10–15 min, and further incubated with anti-β-catenin (1:500; Proteintech) overnight in a humid chamber at 4 °C. Lastly, the coverslips were co-incubated with FITC-conjugated goat anti-rabbit IgG (1:100; Proteintech) at 37 °C for 1 h in the dark, sealed with anti-fluorescence quenching sealing liquid (including DAPI; Beyotime Biotech), and imaged using a fluorescence microscope (Ni-U, Nikon, Tokyo, Japan).

### ART sensitivity assay of PP-treated CAL-62 cells

To evaluate the effects of PP on CAL-62 cells, a CCK-8 cell proliferation assay was performed in 96-well plates. CAL-62 cells were treated with or without 50, 60, 70, 80, 90, 100, 150, and 200 nM PP for 48 h. To evaluate the response of CAL-62 cells to the combination of ART and PP, a CCK-8 cell proliferation assay was performed. CAL-62 cells were treated with a combination of 150 μM ART and 60, 70, 80, 90, and 100 nM PP, or a combination of 80 nM PP and 50, 100, 150, and 200 μM ART for 48 h. CAL-62 cells treated with 0.2% DMSO were used as the control and CAL-62 cells treated with or without 80 nM PP combined with 150 μM ART for 48 h were used for flow cytometry. Protein samples for western blotting were obtained from CAL-62 cells treated with a combination of 80 nM PP and 150 μM ART, or 0.2% DMSO for 48 h. Flow cytometry and western blotting were performed using previously published reports [12]. A DNA fragmentation assay was performed using CAL-62 cells on cell-bearing coverslips (after treatment with 150 μM ART and 80 nM PP for 48 h) by using the terminal deoxynucleotide transferase-mediated dUTP-biotin nick end-labeling method (TUNEL; Proteintech). Cells on coverslips with 0.2% DMSO were used as a negative control. Fluorescence microscopy (Ni-U, Nikon) was used to observe and photograph the cells on coverslips.

### Statistical analysis

Each experiment was performed three times and the data were presented as the mean. The data obtained from the CCK-8 cell proliferation assay was evaluated using independent samples *t*-test and ANOVA. Data in the bar graphs represent the mean ± standard deviation (SD) of individual experiments (*n* ≥ 5). The *p*-values are indicated in the figures and their legends.

## Results

### ART suppresses the growth of BHT-101 and resistance in CAL-62 cells

**Fig. 1:**
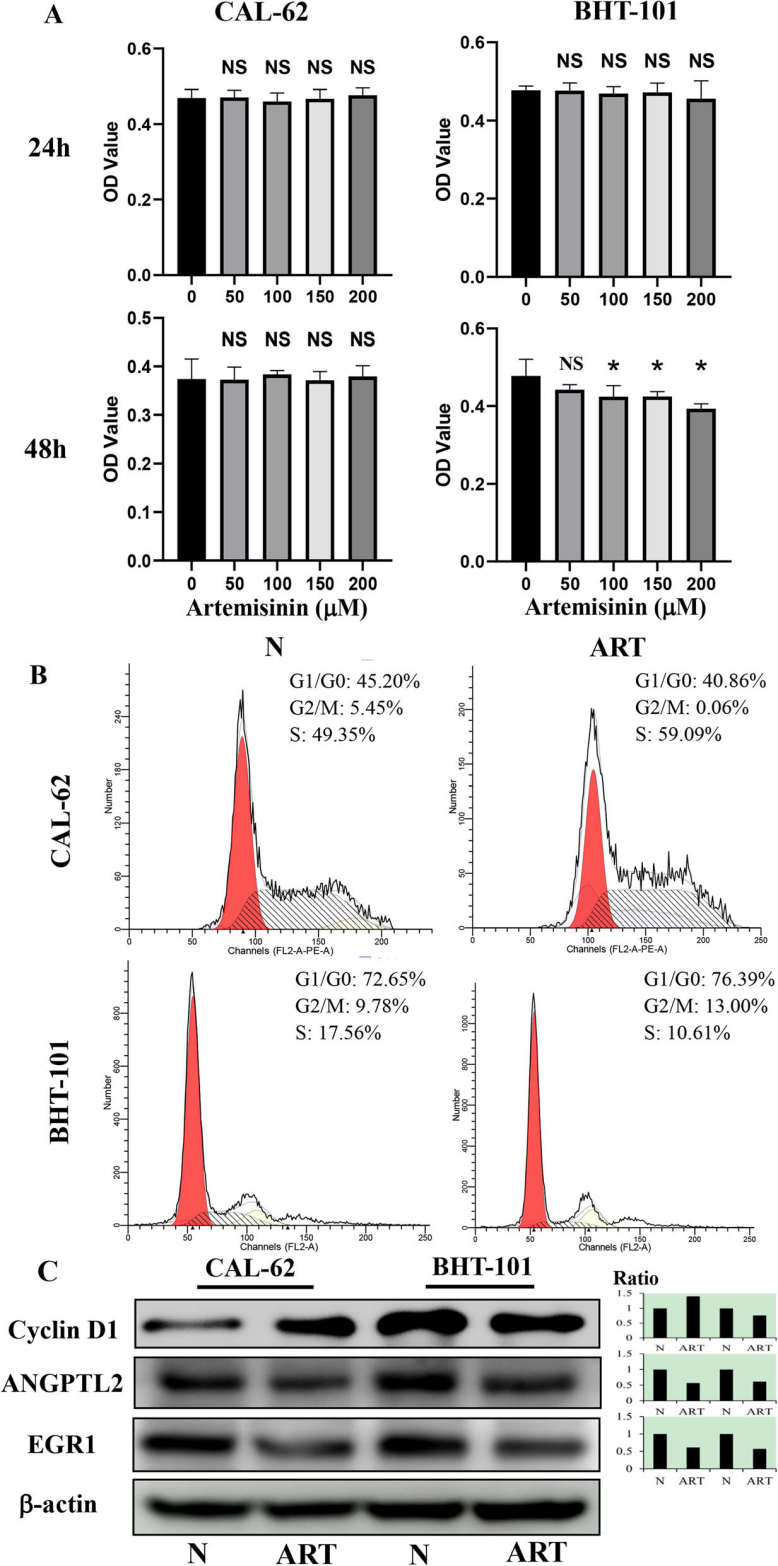
Effects of artemisinin (ART) on anaplastic thyroid cancer. **A** CCK-8 proliferation assay after ART treatment for 24 and 48 h. **B** Flow cytometry of cells treated with or without 100 μM ART for 48 h. **C** Western blotting of cyclin D1, ANGPTL2, and EGR1 after treatment with 100 μM ART for 48 h. *, statistical significance (*p* < 0.05); NS, not significant (*p* > 0.05); error bars, mean ± standard deviation; ratio, the ratio of integrated density (treatment and untreated cells). Gels/blots are cropped for a clear presentation of results. Protein samples derived from the same experiment and gels/blots were processed in parallel. N, groups that did not receive ART treatment. ART, groups that received 100 μM ART treatment. Red, G1/G0; yellow, G2/M; slash, S

Results of the CCK-8 proliferation assay (Fig. 1A) revealed that the OD values of BHT-101 cells decreased significantly in a dose-dependent manner (*p* < 0.05) after treatment with 100, 150, and 200 μM ART for 48 h compared with the control group treated with 0.2% DMSO. When the assay was performed after treatment for 24 h, ART did not show any significant growth inhibition of BHT-101 cells. Results from flow cytometry (Fig. 1B) revealed a majority of cells at the G2/M phase (9.78 to 13.00%) and a reduction of cells in the S phase (17.56 to 10.61%) in BHT-101 cells treated with 100 μM ART for 48 h. No significant differences (*p* > 0.05) were observed in the OD values (Fig. 1A) of CAL-62 compared with the control cells treated with 0.2% DMSO, or compared with cells treated with ART for 24 h and 48 h. Results of the flow cytometry analysis also showed a reduction in the G2/M phase (5.45 to 0.06%) and an increase of cells in the S phase (49.35 to 59.09%) when CAL-62 cells were treated with 100 μM ART for 48 h. ANGPTL2 expression is associated with a poor prognosis; it can increase the proliferation of thyroid cancer cells and promote their migration and invasion [13]. Changes in the expression of EGR1 can affect proliferation, apoptosis, and activation of immune cells [14]. Using western blotting (Fig. 1C), we found that the expression of EGR1 and ANGPTL2 decreased after CAL-62 and BHT-101 were treated with 100 μM ART. Moreover, cyclin D1 levels decreased in BHT-101 cells and increased in CAL-62 cells, indicating that the growth of BHT-101 cells was suppressed when treated with ART at a concentration of 100 μM or higher, and that ART could not influence the growth of CAL-62 cells.

### Network analysis of mRNA expression between ART-treated and Control groups

**Fig. 2:**
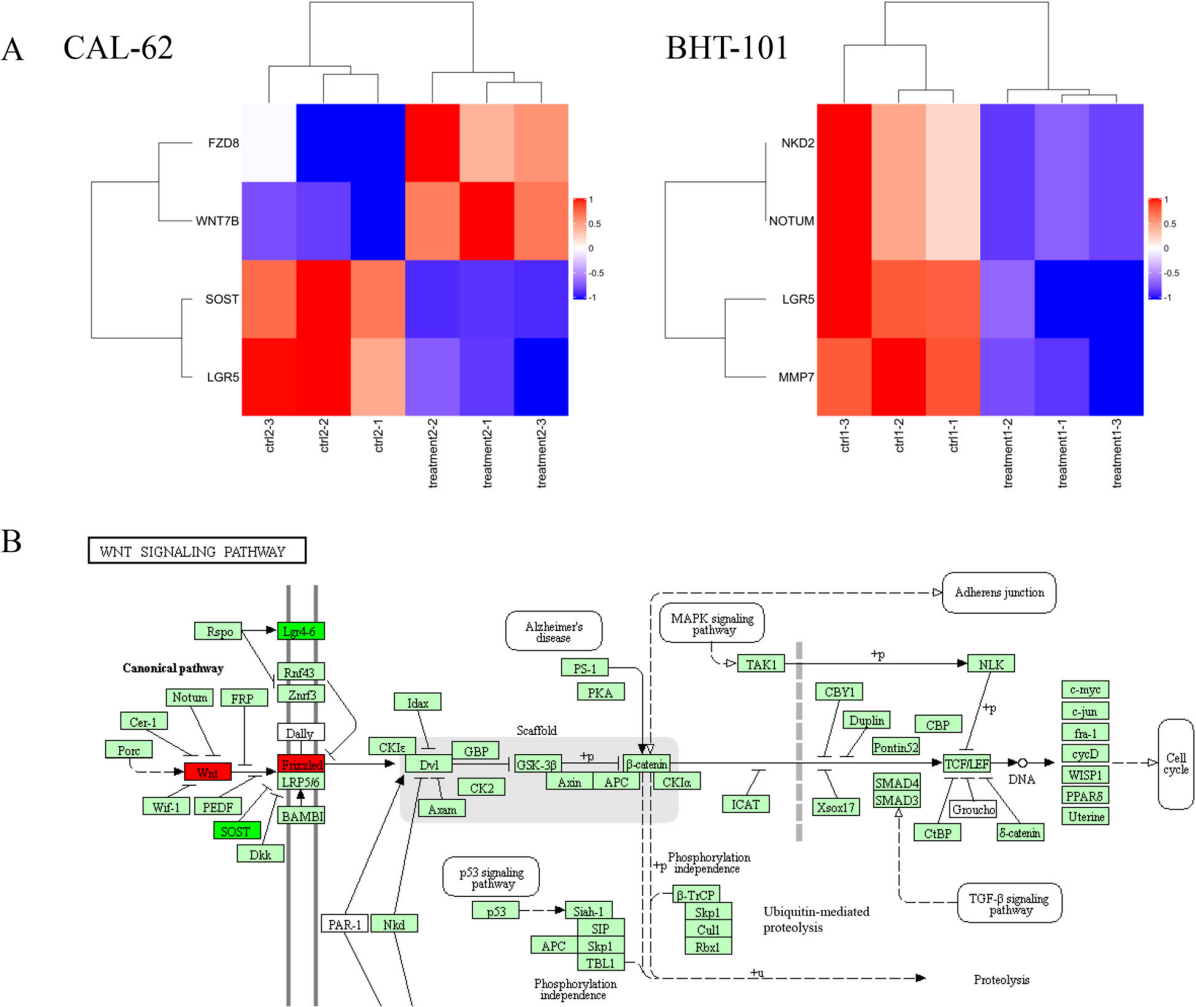
Network analysis of mRNA expression in 100 μM artemisinin-treated and control groups for 48 h. **A** The heatmap of CAL-62 and BHT-101 about targets in the WNT signaling pathway. Red in Fig. 2A means higher expression of mRNA and blue means lower expression of mRNA. **B** The WNT signaling pathway map from KEGG and the targets changed in CAL-62. Red and green colors in Fig. 2B indicate upregulation and downregulation, respectively

Results of the network analysis of mRNA expression showed that after treatment with 100 μM ART for 48 h, LGR5 decreased in CAL-62 and BHT-101 cells; NKD2, NOTUM, and MMP7 decreased in BHT-101 cells; SOST decreased in CAL-62 cells. WNT7B and FZD8 increased after treatment of CAL-62 cells with 100 μM ART for 48 h (Fig. 2A). The roles of WNT7B, FZD, LGR5, and SOST in the WNT signaling pathway are shown in Fig. 2B. The results suggested the upregulation of protein expression of WNT7B and FZD8 in CAL-62 cells after treatment with 100 μM ART for 48 h, and that the WNT signaling pathway might be associated with ART resistance of CAL-62 cells.

### Significant activation of WNT signaling in ART-treated CAL-62 cells

**Fig. 3:**
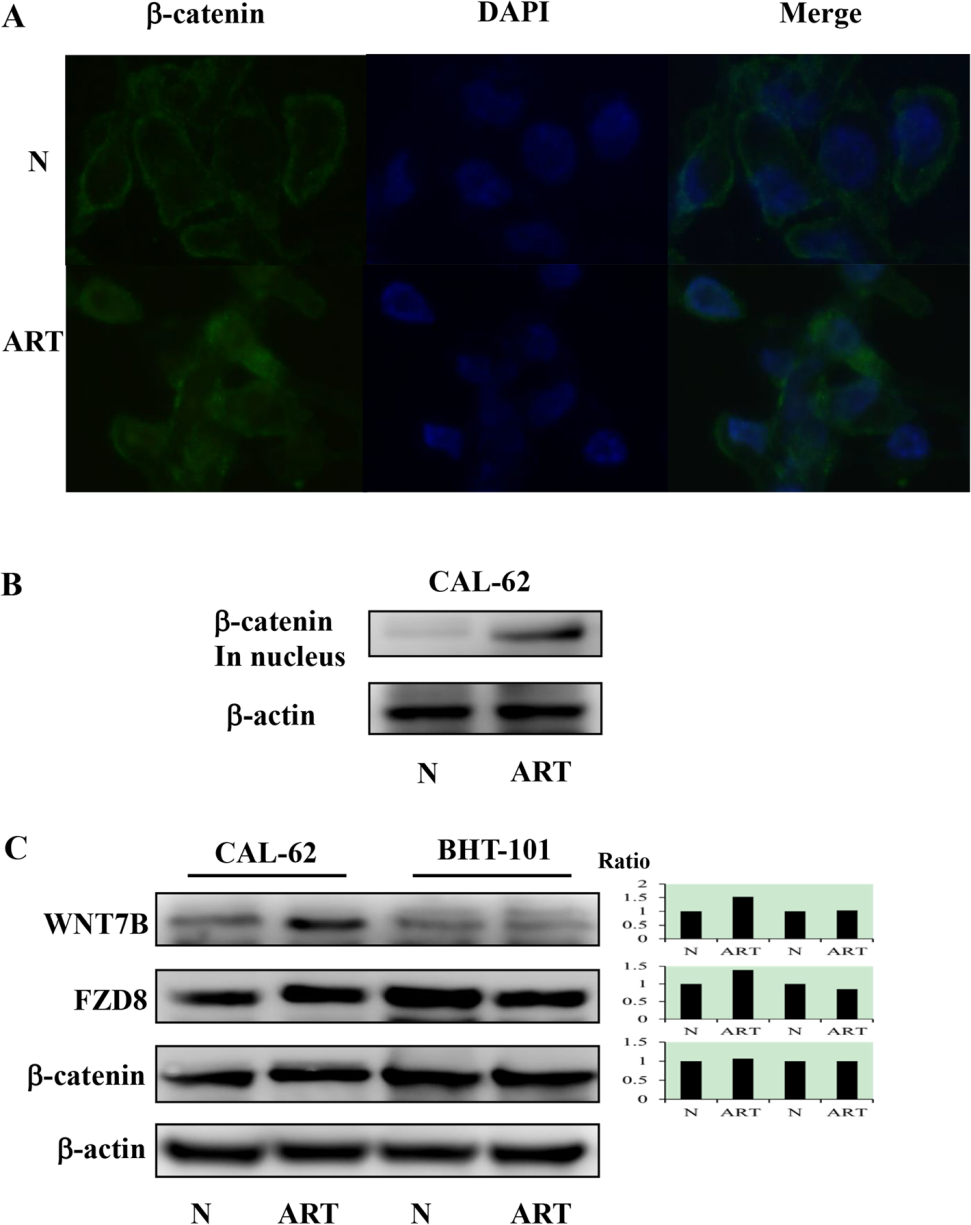
Activation of WNT signaling in 100 μM artemisinin (ART)-treated CAL-62 cells after 48 h. **A** Immunofluorescence labeling (× 40). Western blots of β-catenin in the nuclei. **C** Western blots of WNT7B, FZD8, and β-catenin. Gels/blots are cropped for the clear presentation of results. Samples derived from the same experiment and gels/blots were processed in parallel. Ratio, the ratio of integrated density (treatment and untreated cells); N, groups without ART treatment; ART, groups treated with 100 μM ART

The expression of β-catenin, a target of the canonical WNT signaling pathway, was examined using immunofluorescence labeling to determine WNT signaling. As shown in Fig. 3A, β-catenin is stably expressed in the cytoplasm of CAL-62 cells and distinctly increased in the nuclei after treatment with 100 μM ART for 48 h. Results from western blotting revealed an increase in the expression of β-catenin in the nuclei (Fig. 3B). Moreover, the expression of WNT7B, FZD8, and β-catenin in the cytoplasm was distinctly increased in CAL-62 cells and was found to either decrease slightly or remain unchanged in BHT-101 cells after treatment with 100 μM ART for 48 h (Fig. 3C). Collectively, our findings suggested that the resistance of CAL-62 to ART was related to the upregulation of the WNT signaling pathway.

### Combination of the WNT inhibitor and ART overcomes ART resistance in CAL-62 cells

**Fig. 4:**
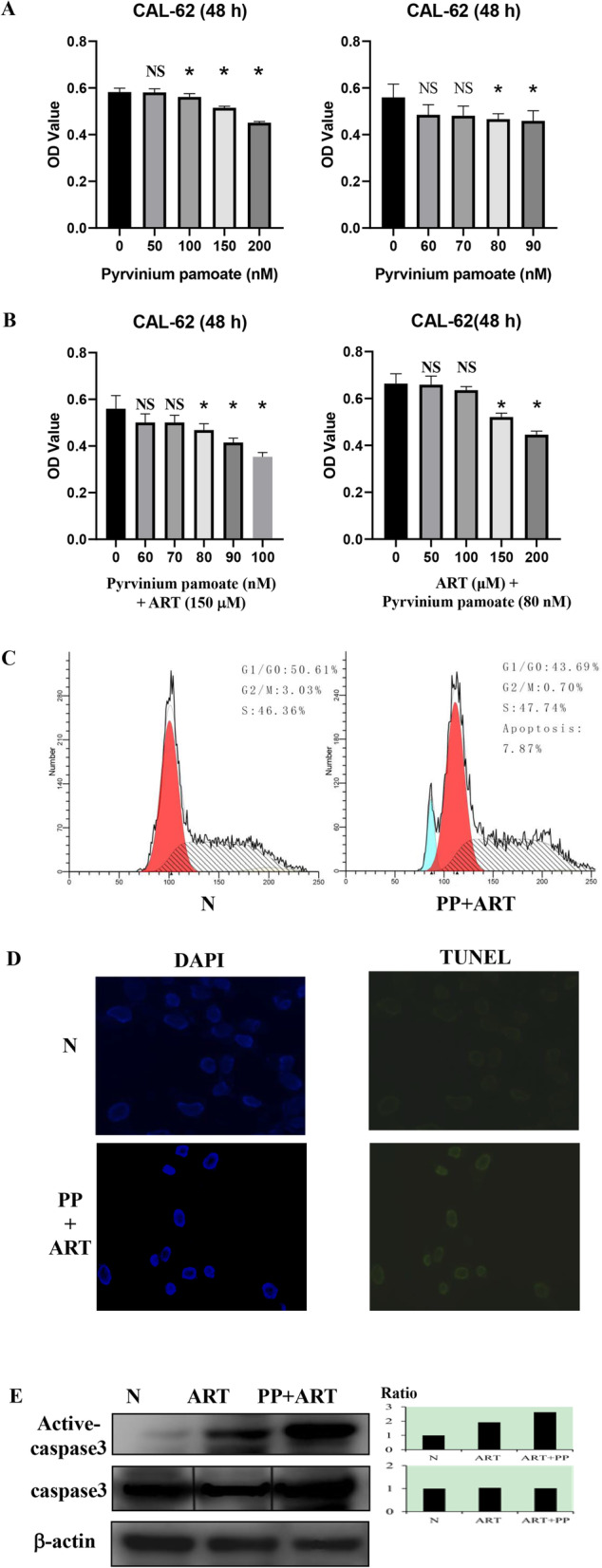
Combination of the WNT inhibitor and artemisinin (ART) could overcome the resistance of CAL-62 cells. **A** CCK-8 proliferation assay of pyrvinium pamoate (PP) treatment alone for 48 h; **B** CCK-8 proliferation assay of cells treated with a combination of PP and ART for 48 h; **C** Flow cytometry of CAL-62 cells treated with or without 80 nM PP combined with 150 μM ART for 48 h; **D** TUNEL assay of CAL-62 cells treated with or without 80 nM PP combined with 150 μM ART for 48 h; **E** Western blotting of active caspase-3 and caspase-3 after treatment with a combination of 80 nM PP and 150 μM ART for 48 h. Gels/blots are cropped for a clear presentation of results. Protein samples derived from the same experiment and gels/blots were processed in parallel. Black line delineates the boundary between gels. Ratio, the ratio of integrated density (treatment and untreated cells); N, groups that were not treated with ART; ART, groups treated with 150 μM ART; PP + ART, groups treated with a combination of 80 nM PP and 150 μM ART. Blue, apoptosis; red, G1/G0; yellow, G2/M; slash, S

After treatment with 80, 90, 100, 150, and 200 nM of the WNT inhibitor, PP, for 48 h, the OD values of CAL-62 decreased significantly (*p* < 0.05) in a dose-related manner compared with the group treated with 0.2% DMSO (Fig. 4A). The groups treated with a combination of 150 μM ART and 80, 90, or 100 nM PP showed a significant decrease (*p* < 0.05) in OD value in a dose-dependent manner compared with those treated with 0.2% DMSO for 48 h (Fig. 4B). Moreover, treatment with the combination of 80 nM PP and 150 and 200 μM ART for 48 h led to a dose-dependent and significant decrease (*p* < 0.05) in OD compared with the group treated with 0.2% DMSO. Results from flow cytometry revealed a distinct increase in the apoptosis index (7.87%) and the presence of TUNEL-positive cells compared with cells treated with 0.2% DMSO (Figs. 4C and D). The findings from western blotting indicated that the levels of active caspase-3 increased after treatment with 80 nM PP and 150 μM ART (Fig. 4E). Collectively, these findings suggested that CAL-62 cells might resist the antitumor effects of ART by the activation of WNT signaling, and that combination therapy with PP and ART could suppress the growth of CAl-62 cells and induce apoptosis through a considerable increase of active caspase-3.

## Discussion

Although ATC has a low incidence, it is the most lethal variant of TC with a 1 year survival for most patients [15]. ART is a compound obtained from natural sources. It exerts low toxicity in normal cells and is known to generate reactive oxygen species (ROS) and induce apoptosis [10]. To the best of our knowledge, there are no published data regarding the effects of ART in ATC.

In this study, BHT-101 and CAL-62 cells were found to have different sensitivities to the same concentration of ART. Our results revealed distinct inhibitory effects of 100 μM ART on BHT-101 cells, as well as cell cycle arrest (G1: 72.35 to 76.39%; G2: 9.78 to 13.00%) with downregulation of cyclin D1, suggesting the therapeutic value of ART in the management of ATC. ART is not used as an anti-ATC agent and similar antitumor effects were not observed when CAL-62 cells were treated with this compound under identical experimental conditions. Although the expression of proliferation markers including ANGPTL2 and EGR1 was decreased in CAL-62 and BHT-101 cells, the inhibitory effects of ART in CAL-62 cells were not significant, suggesting the requirement of personalized anti-ATC therapy using ART. There is an urgent need to develop therapeutic approaches for the treatment of ATC cells. Thus, it would be worthwhile to elucidate the specific molecular mechanisms underlying the effects of ART in CAL-62 and BHT-101 cells.

Many patients may experience a relapse and progression of cancer because of acquired resistance [16]. To determine the mechanism of resistance of CAL-62 to ART, mRNA sequencing was performed using CAL-62 and BHT-101 cells. Sequencing results showed a distinct increase in the mRNA level of WNT7B and FZD8 after treatment of CAL-62 cells with 100 μM ART, whereas no increase was observed in BHT-101 cells. Thus, we reasoned that WNT signaling activity might be related to ART resistance in CAL-62 cells.

WNT7B is a member of the WNT ligand family and its expression is an important prognostic factor for the overall survival as well as recurrence-free survival of patients with breast cancer [17]. FZD8 is a target of p53, which is related to the activation of the canonical WNT/β-catenin signaling pathway [18]. WNT7B and FZD8 are the ligand and receptor of the WNT signaling pathway. The WNT pathway can be divided into the canonical (β-catenin dependent) and non-canonical (β-catenin independent) signaling pathways. The canonical WNT signaling pathway likely plays a role in the development of tumors, and the development of gastrointestinal cancers, leukemia, melanoma, and breast cancer [19]. We found that the expression of β-catenin, FZD8, and WNT7B increased after the treatment of CAL-62 cells with 100 μM ART. Especially, the expression of β-catenin in the nucleus increased significantly, which confirmed the activation of β-catenin after the treatment of CAL-62 cells with 100 μM ART. The constitutive activation of the WNT/β-catenin signaling pathway leads to the increased expression of cyclin D1, Myc, and other essential proteins related to cell proliferation and cell-cycle progression [20]. Collectively, these findings suggested that the activity of the canonical WNT signaling pathway was associated with the effects of ART in CAL-62 cells.

Pyrvinium pamoate is an FDA-approved inhibitor of the WNT signaling pathway. It significantly decreases the expression of FZD1, FZD10, WNT1, and WNT7B at the transcriptional level [21]. In our study, we found that 100 nM PP could suppress the growth of CAL-62 cells for 48 h. The combination of 150 μM ART and 80 nM PP significantly suppressed the proliferation of CAL-62 cells and showed an increased number of TUNEL-positive cells, indicating the apoptotic fraction with distinct caspase-3 activation. Based on these findings, it would be reasonable to conclude that CAL-62 cells overcome ART resistance by an increase in WNT signaling and the upregulation of WNT signaling activity. ART resistance in ATC cells depends on the WNT signaling pathway. Therefore, the combination of PP and ART would be of potential value in improving drug efficacy and avoiding ART resistance in the management of ATC. Future studies should be targeted at obtaining more clinical evidence of the activity of anticancer drugs in ATC through upregulated WNT signaling activity. Moreover, the specific mechanism of action and molecular targets involved when a combination of PP and ART is used should be further clarified.

## Conclusions

To summarize, we have highlighted the efficacy of ART either as monotherapy or in combination with PP. By showing the association between ART resistance and WNT signaling activity, we have presented a novel target that can be used to overcome drug resistance in ATC.. However, these effects still need to be further demonstrated in vivo.

## Supplementary Information

**Additional file 1: Figure 1.1.** The Uncropped Blot of Cyclin D1 (34kDa)-1. The red arrow indicates the location of target bands. **Figure 1.2.** The Uncropped Blot of Cyclin D1 (34kDa)-2. The red arrow indicates the location of target bands. **Figure 1.3.** The Uncropped Blot of Cyclin D1 (34kDa)-3. The red arrowindicates the location of target bands**. Figure 2.1.** The Uncropped Blot of ANGPTL2 (57 kDa)-1. The red arrow indicates the location of target bands. **Figure 2.2.** The Uncropped Blot of ANGPTL2 (57 kDa)-2. The red arrow indicates the location of target bands. **Figure 2.3.** The Uncropped Blot of ANGPTL2 (57 kDa)-3. The red arrow indicates the location of target bands. **Figure 3.1.** The Uncropped Blot of EGR1 (56 kDa)-1. The red arrow indicates the location of target bands. **Figure 3.2.** The Uncropped Blot of EGR1 (56 kDa)-2. The red arrow indicates the location of target bands. **Figure 3.3.** The Uncropped Blot of EGR1 (56 kDa)-3. The red arrow indicates the location of target bands. **Figure 4.** The Uncropped Blot of β-actin (42 kDa). The red arrow indicates the location of target bands. **Figure 5.1.** The Uncropped Blot of β-catenin (92 kDa) in Nucleus-1. The red arrow indicates the location of target bands. **Figure 5.2.** The Uncropped Blot of β-catenin (92 kDa) in Nucleus-2. The red arrow indicates the location of target bands. **Figure 5.3.** The Uncropped Blot of β-catenin (92 kDa) in Nucleus-3. The red arrow indicates the location of target bands. **Figure 6.** The Uncropped Blot of β-actin (42 kDa). The red arrow indicates the location of target bands. **Figure 7.1.** The Uncropped blot of β-catenin (92 kDa)-1. The red arrow indicates the location of target bands. **Figure 7.2.** The Uncropped blot of β-catenin (92 kDa)-2. The red arrow indicates the location of target bands. **Figure 7.3.** The Uncropped blot of β-catenin (92 kDa)-3. The red arrow indicates the location of target bands. **Figure 8.1.** The Uncropped blot of WNT7B (39 kDa)-1. (A) The Origin Picture of β-catenin. The red arrow indicates the location of target bands. (B) The Marker of β-catenin. (C) The Merge of Blot and Marker. **Figure 8.2.** The Uncropped blot of WNT7B (39 kDa)-2. The red arrow indicates the location of target bands. **Figure 8.3.** The Uncropped blot of WNT7B (39 kDa)-3. The red arrow indicates the location of target bands. **Figure 9.1.** The Uncropped blot of FZD8 (60 kDa)-1. The red arrow indicates the location of target bands. **Figure 9.2.** The Uncropped blot of FZD8 (60 kDa)-2. The red arrow indicates the location of target bands. **Figure 9.3.** The Uncropped blot of FZD8 (60 kDa)-3. The red arrow indicates the location of target bands. **Figure 10.** The Uncropped Blot of β-actin (42 kDa). The red arrow indicates the location of target bands. **Figure 11.1.** The Uncropped blot of Phospho-Caspase 3 (34 kDa)-1. (A) The Origin Picture of Phospho-Caspase 3. The red arrow indicates the location of target bands. (B) The Marker of Phospho-Caspase 3. (C) The Merge of Blot and Marker. **Figure 11.2.** The Uncropped blot of Phospho-Caspase 3 (34 kDa)-2.The red arrow indicates the location of target bands. **Figure 11.3.** The Uncropped blot of Phospho-Caspase 3 (34 kDa)-3.The red arrow indicates the location of target bands. **Figure 12.1.** The Uncropped Blot of Caspase 3 (37 kDa)-1. The red arrow indicates the location of target bands. **Figure 12.2.** The Uncropped Blot of Caspase 3 (37 kDa)-2. The red arrow indicates the location of target bands. **Figure 12.3.** The Uncropped Blot of Caspase 3 (37 kDa)-3. The red arrow indicates the location of target bands. **Figure 13.** The Uncropped Blot of β-actin (42 kDa). The red arrow indicates the location of target bands.

## Data Availability

The datasets used and/or analyzed during the current study are available from the corresponding author on reasonable request.

## Abbreviations

ART: Artemisinin
PP: Pyrvinium pamoate
TC: Thyroid cancer
ATC: Anaplastic thyroid cancer
FBS: Fetal bovine serum
DMSO: Dimethyl-sulfoxide
TUNEL: Terminal deoxynucleotide transferase-mediated dUTP-biotin nick end-labeling method
