# Supplementary material for: Pyrvinium pamoate can overcome artemisinin’s resistance in anaplastic thyroid cancer
Source: BMC Complement Med Ther. 2021 May 28;21:156. doi: 10.1186/s12906-021-03332-z (PMC8161585; doi:10.1186/s12906-021-03332-z)
Supplement: Supplementary file 1 — Additional file 1: Figure 1.1. The Uncropped Blot of Cyclin D1 (34kDa)-1. The red arrow indicates the location of target bands. Figure 1.2. The Uncropped Blot of Cyclin D1 (34kDa)-2. The red arrow indicates the location of target bands. Figure 1.3. The Uncropped Blot of Cyclin D1 (34kDa)-3. The red arrowindicates the location of target bands. Figure 2.1. The Uncropped Blot of ANGPTL2 (57 kDa)-1. The red arrow indicates the location of target bands. Figure 2.2. The Uncropped Blot of ANGPTL2 (57 kDa)-2. The red arrow indicates the location of target bands. Figure 2.3. The Uncropped Blot of ANGPTL2 (57 kDa)-3. The red arrow indicates the location of target bands. Figure 3.1. The Uncropped Blot of EGR1 (56 kDa)-1. The red arrow indicates the location of target bands. Figure 3.2. The Uncropped Blot of EGR1 (56 kDa)-2. The red arrow indicates the location of target bands. Figure 3.3. The Uncropped Blot of EGR1 (56 kDa)-3. The red arrow indicates the location of target bands. Figure 4. The Uncropped Blot of β-actin (42 kDa). The red arrow indicates the location of target bands. Figure 5.1. The Uncropped Blot of β-catenin (92 kDa) in Nucleus-1. The red arrow indicates the location of target bands. Figure 5.2. The Uncropped Blot of β-catenin (92 kDa) in Nucleus-2. The red arrow indicates the location of target bands. Figure 5.3. The Uncropped Blot of β-catenin (92 kDa) in Nucleus-3. The red arrow indicates the location of target bands. Figure 6. The Uncropped Blot of β-actin (42 kDa). The red arrow indicates the location of target bands. Figure 7.1. The Uncropped blot of β-catenin (92 kDa)-1. The red arrow indicates the location of target bands. Figure 7.2. The Uncropped blot of β-catenin (92 kDa)-2. The red arrow indicates the location of target bands. Figure 7.3. The Uncropped blot of β-catenin (92 kDa)-3. The red arrow indicates the location of target bands. Figure 8.1. The Uncropped blot of WNT7B (39 kDa)-1. (A) The Origin Picture of β-catenin. The red arrow indicates th [file 12906_2021_3332_MOESM1_ESM.pdf]

The Uncropped Blot of Cyclin D1 (34kDa)-1

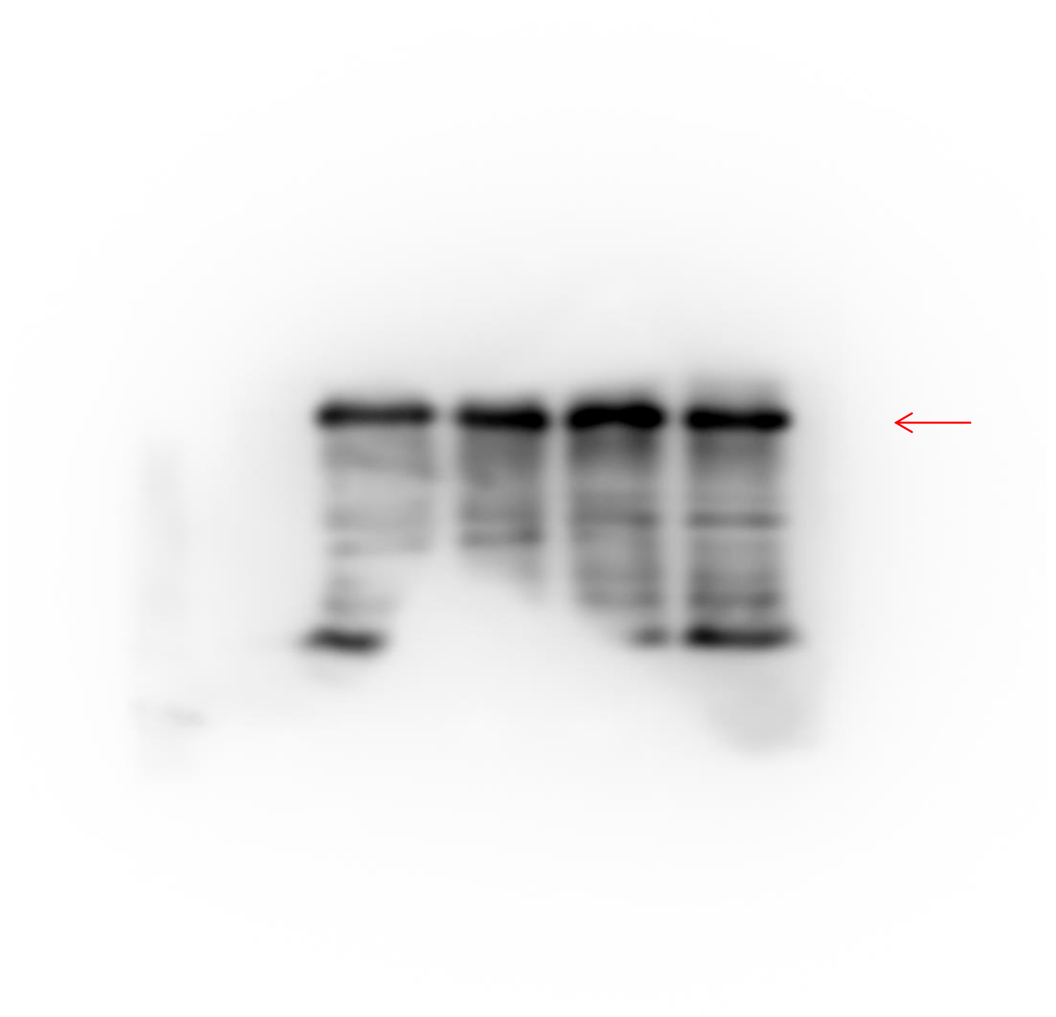

Figure 1.1 The Uncropped Blot of Cyclin D1 (34kDa)-1. The red arrow indicates the location of target bands.

The Uncropped Blot of Cyclin D1 (34kDa)-2

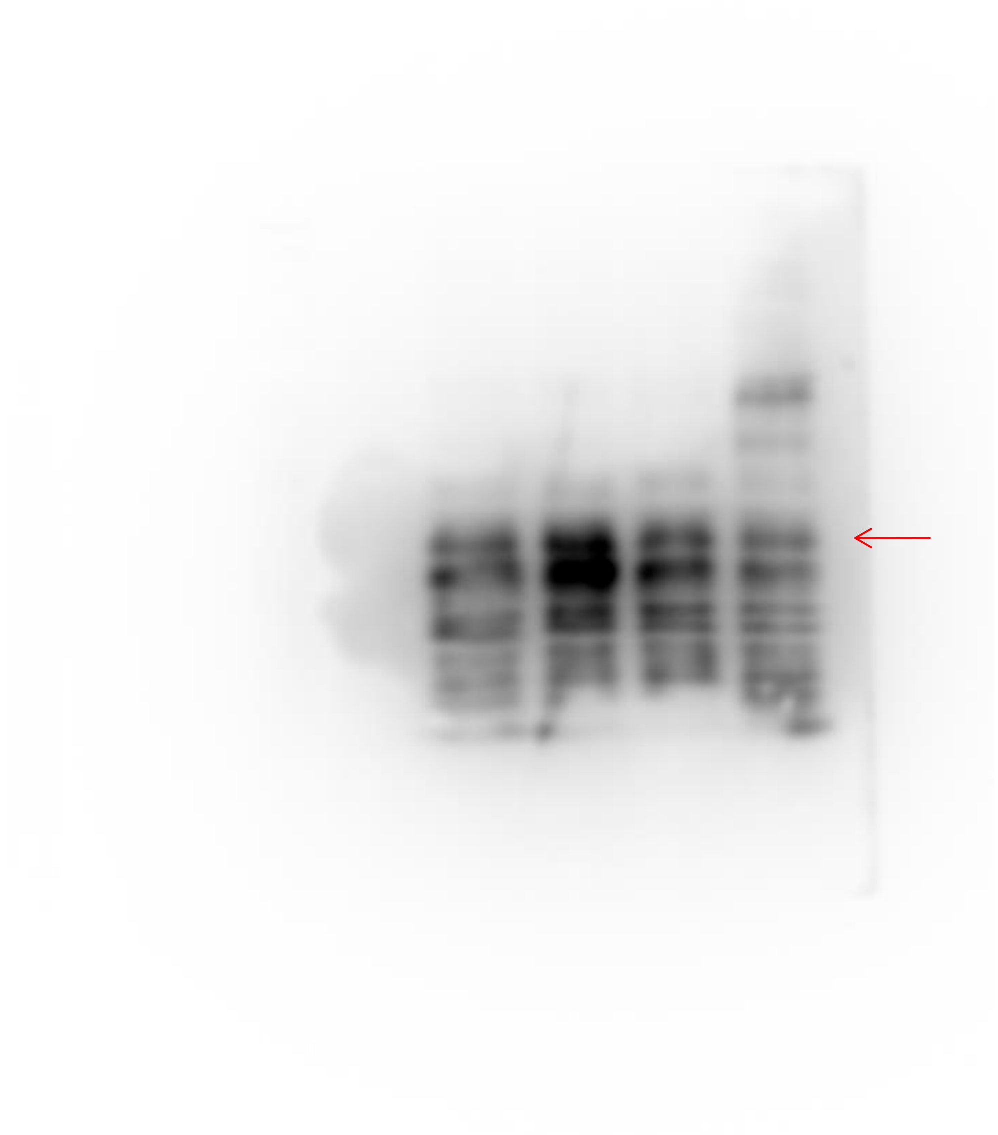

Figure 1.2 The Uncropped Blot of Cyclin D1 (34kDa)-2. The red arrow indicates the location of target bands.

The Uncropped Blot of Cyclin D1 (34kDa)-3

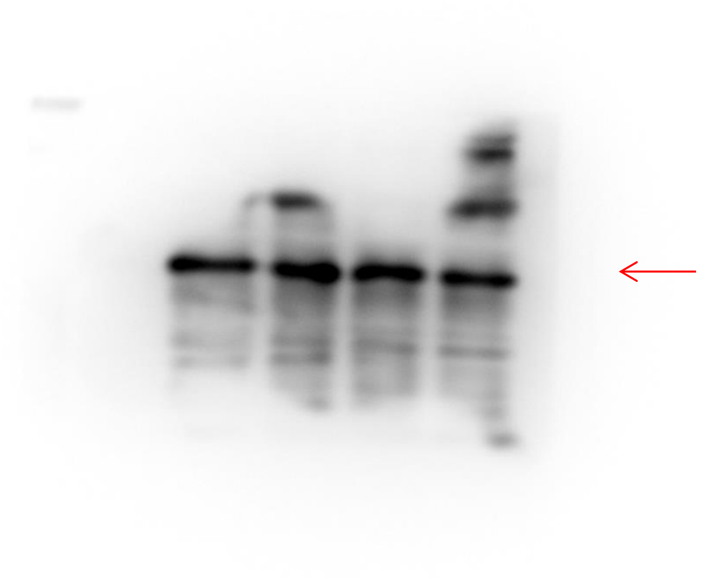

Figure 1.3 The Uncropped Blot of Cyclin D1 (34kDa)-3. The red arrow indicates the location of target bands.

The Uncropped Blot of ANGPTL2 (57kDa)-1

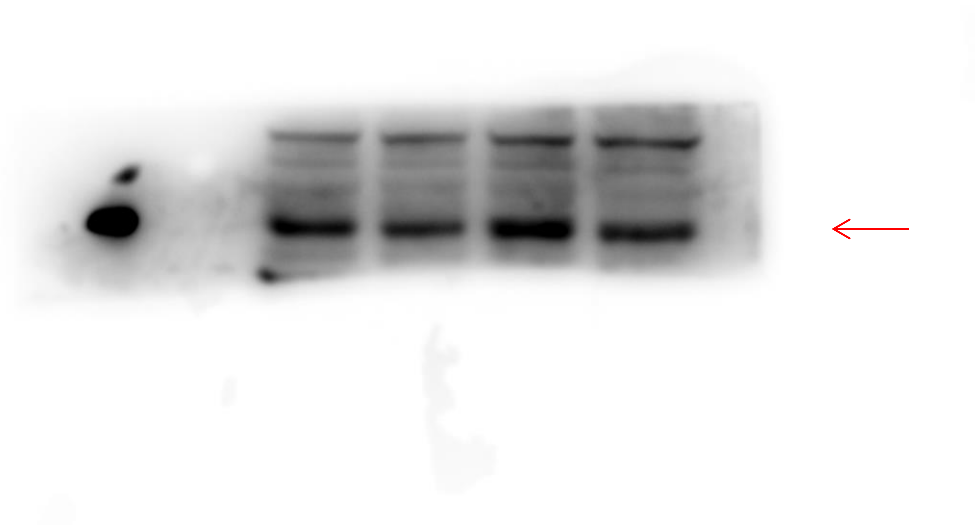

Figure 2.1 The Uncropped Blot of ANGPTL2 (57kDa)-1. The red arrow indicates the location of target bands.

The Uncropped Blot of ANGPTL2 (57kDa)-2

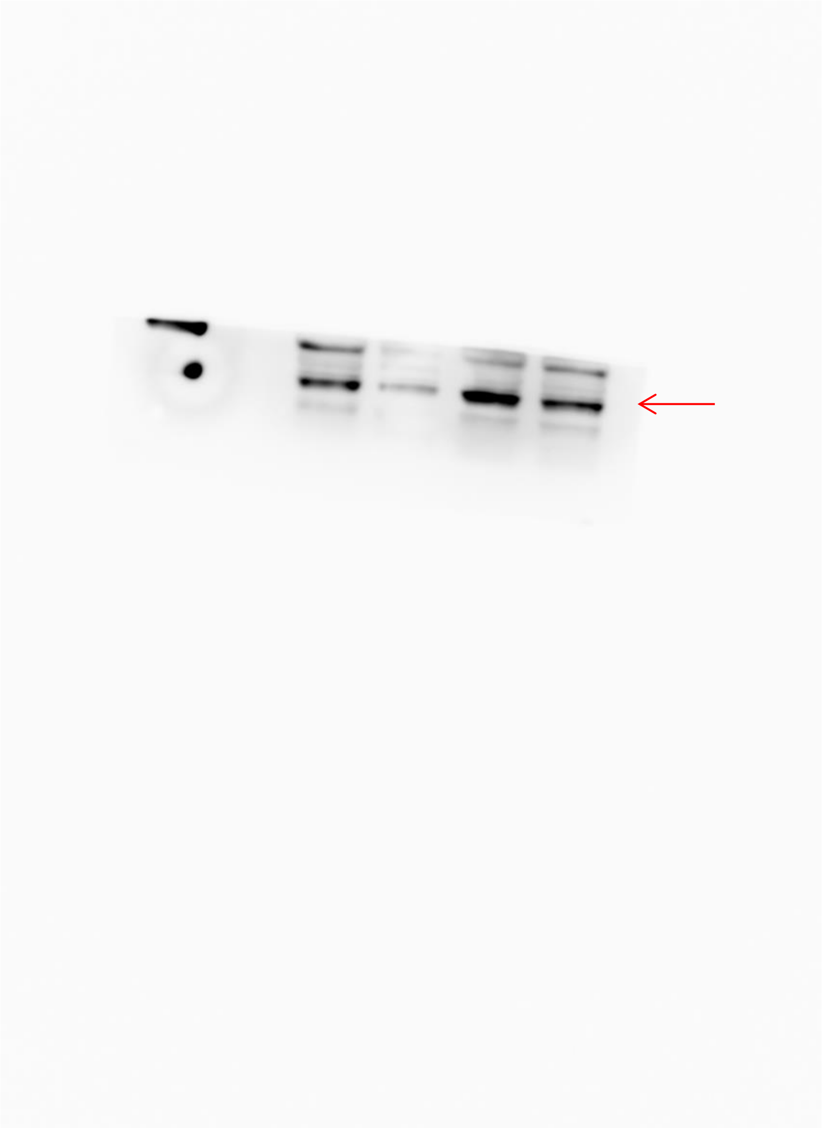

Figure 2.2 The Uncropped Blot of ANGPTL2 (57kDa)-2. The red arrow indicates the location of target bands.

The Uncropped Blot of ANGPTL2 (57kDa)-3

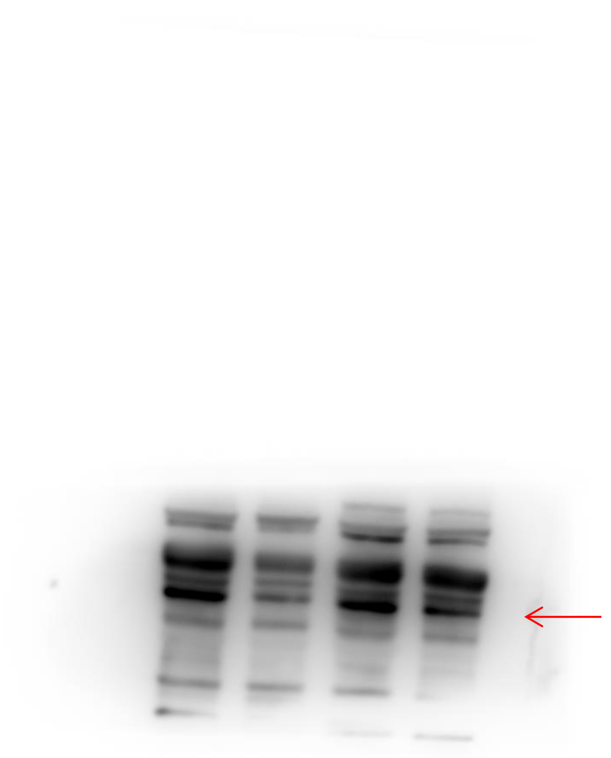

Figure 2.3 The Uncropped Blot of ANGPTL2 (57kDa)-3. The red arrow indicates the location of target bands.

The Uncropped Blot of EGR1 (56kDa)-1

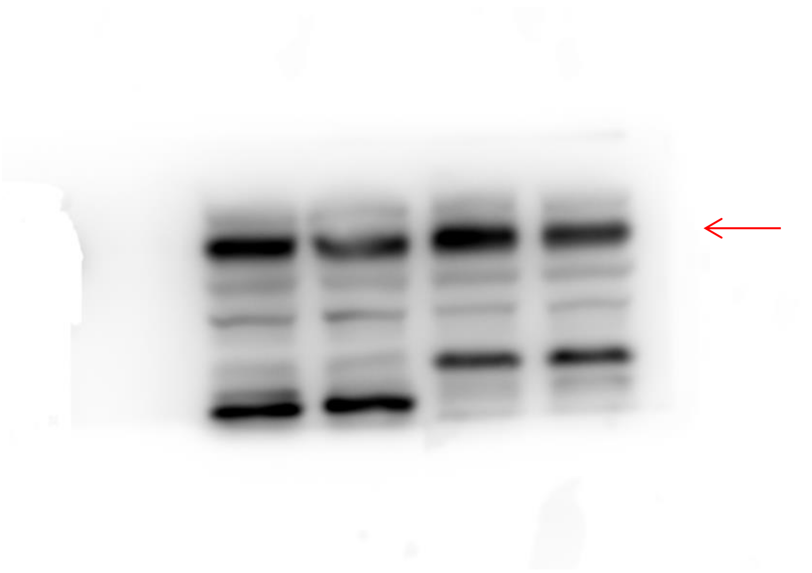

Figure 3.1 The Uncropped Blot of EGR1 (56kDa)-1. The red arrow indicates the location of target bands.

The Uncropped Blot of EGR1 (56kDa)-2

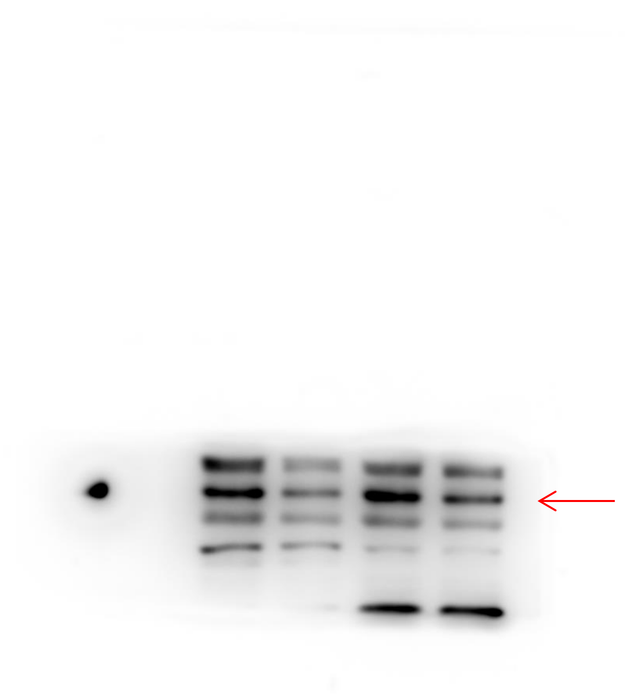

Figure 3.2 The Uncropped Blot of EGR1 (56kDa)-2. The red arrow indicates the location of target bands.

The Uncropped Blot of EGR1 (56kDa)-3

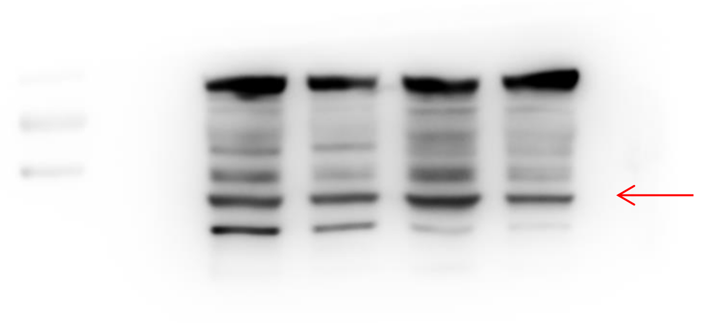

Figure 3.3 The Uncropped Blot of EGR1 (56kDa)-3. The red arrow indicates the location of target bands.

The Uncropped Blot of β-actin (42kDa)

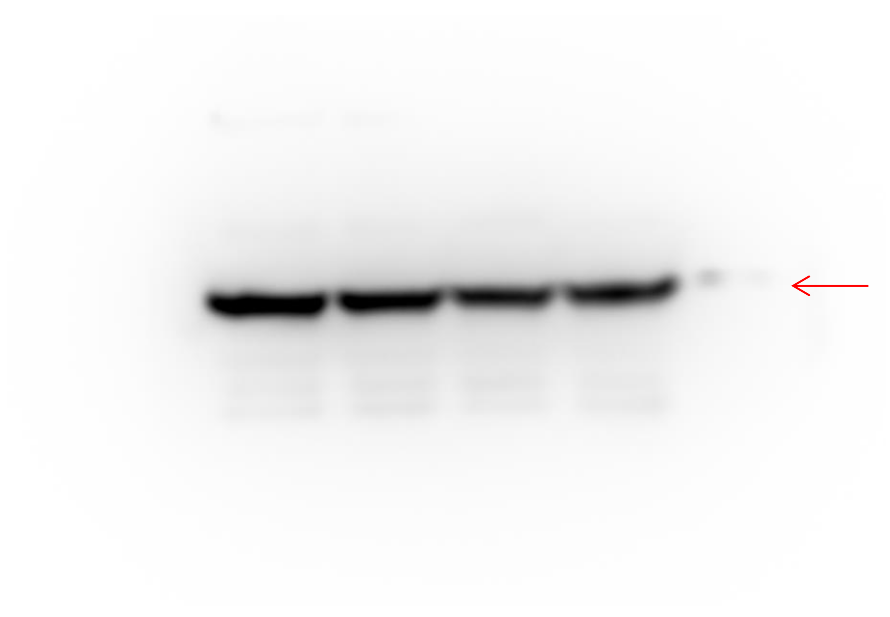

Figure 4 The Uncropped Blot of β-actin (42kDa). The red arrow indicates the location of target bands.

The Uncropped Blot of β-catenin (92kDa) in Nucleus-1

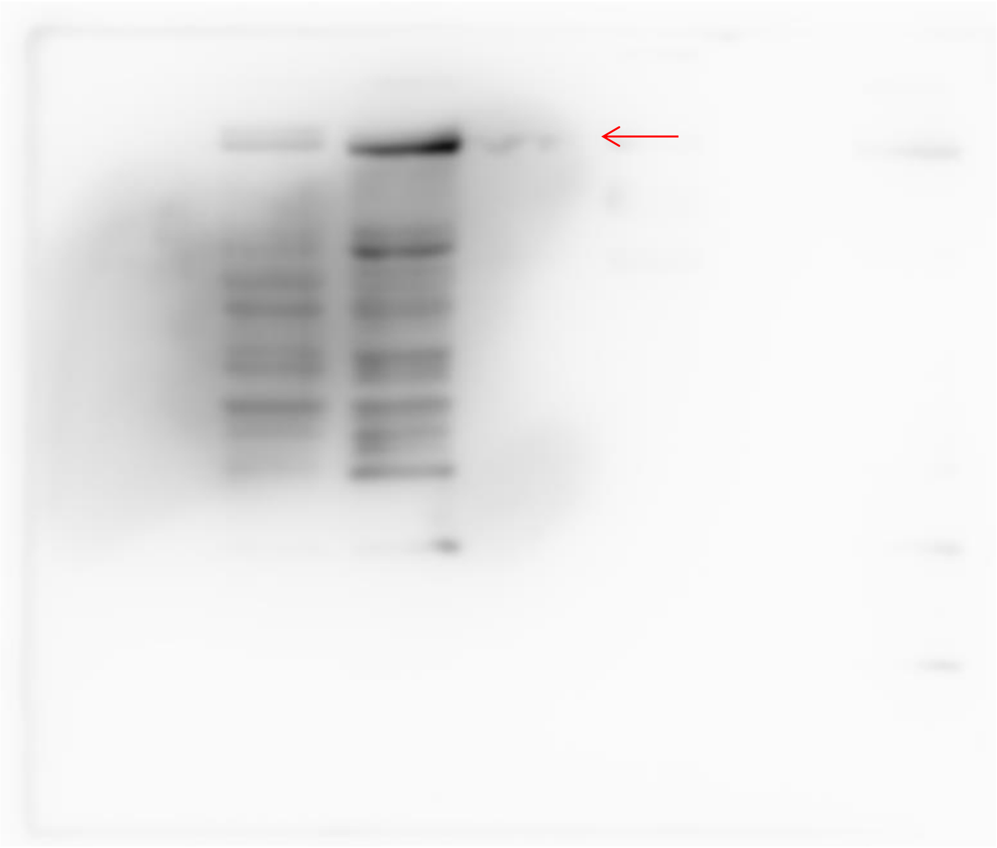

Figure 5.1 The Uncropped Blot of β-catenin (92kDa) in Nucleus-1. The red arrow indicates the location of target bands.

The Uncropped Blot of β-catenin (92kDa) in Nucleus-2

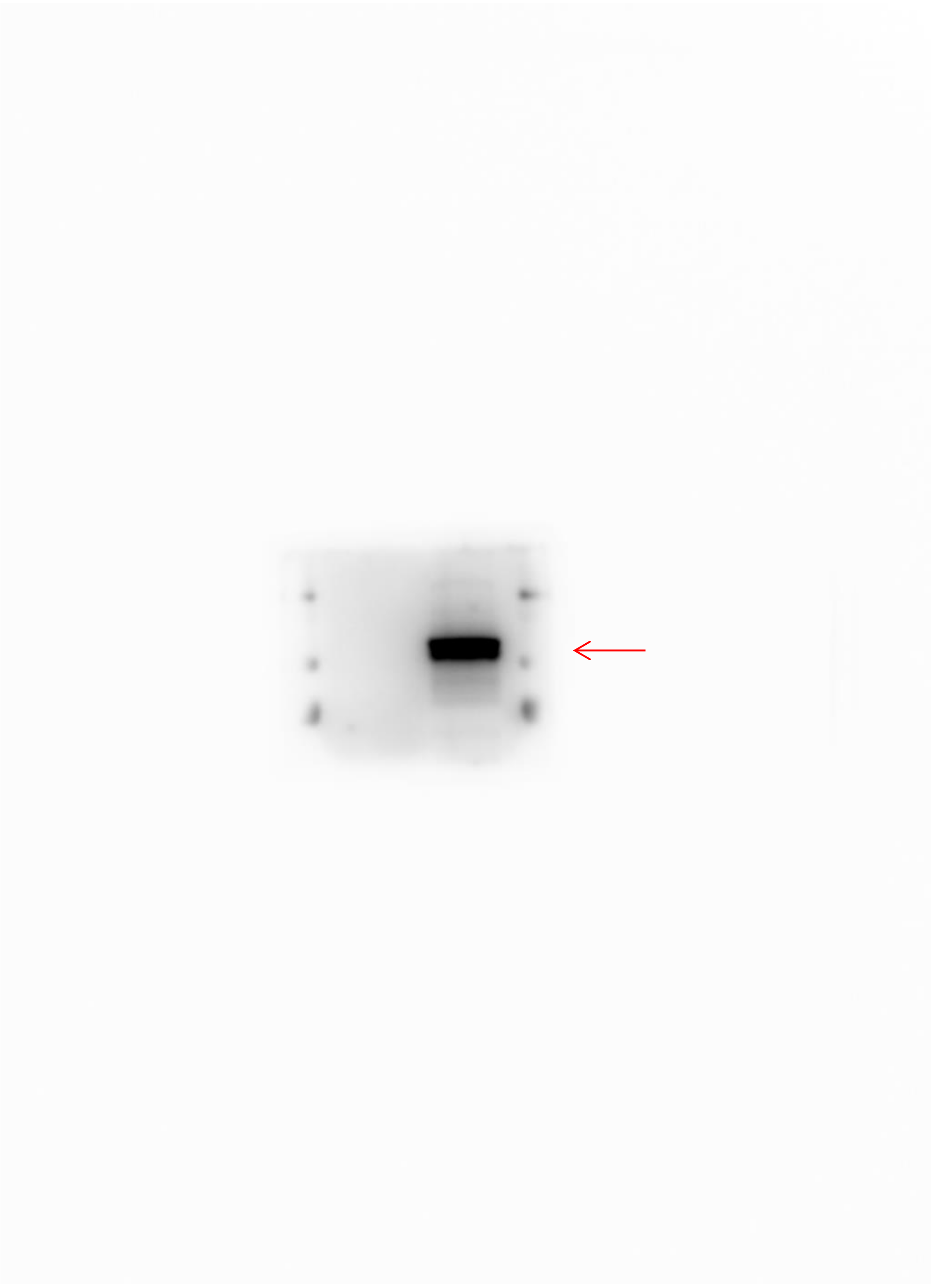

Figure 5.2 The Uncropped Blot of β-catenin (92kDa) in Nucleus-2. The red arrow indicates the location of target bands.

The Uncropped Blot of β-catenin (92kDa) in Nucleus-3

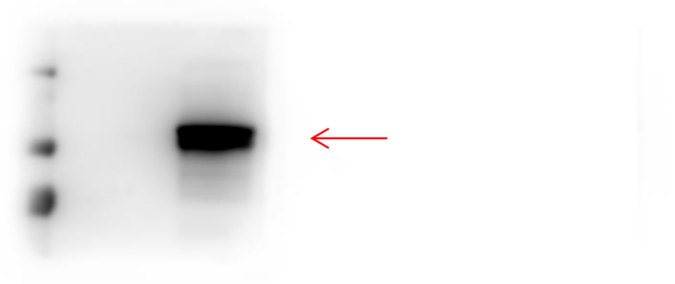

Figure 5.3 The Uncropped Blot of β-catenin (92kDa) in Nucleus-3. The red arrow indicates the location of target bands.

The Uncropped Blot of β-actin (42kDa)

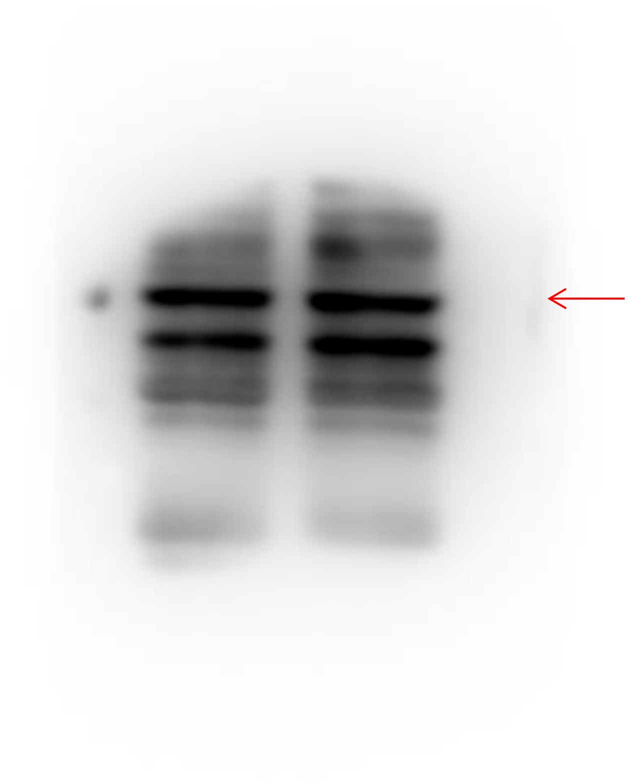

Figure 6 The Uncropped Blot of β-actin (42kDa). The red arrow indicates the location of target bands.

The Uncropped blot of β-catenin (92kDa)-1

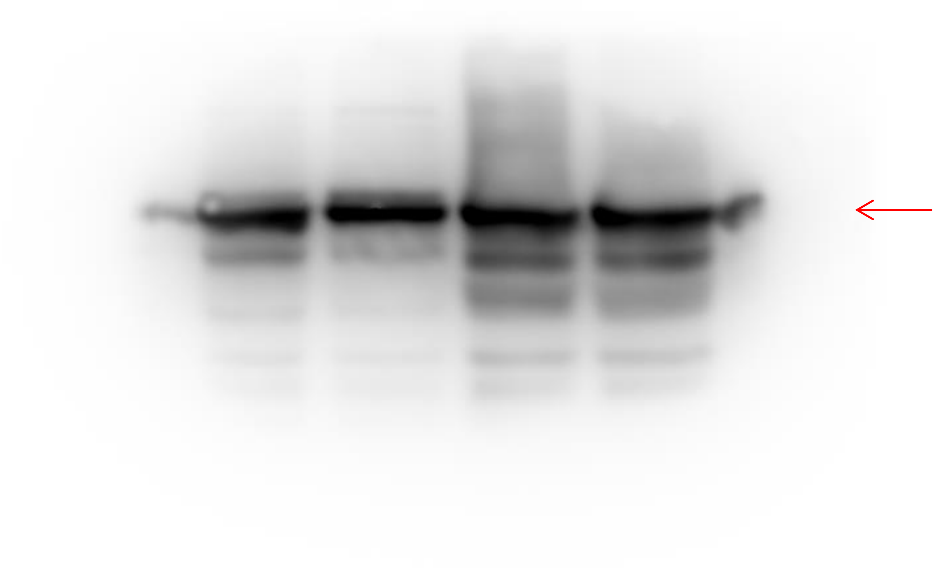

Figure 7.1 The Uncropped blot of β-catenin (92kDa)-1. The red arrow indicates the location of target bands.

The Uncropped blot of β-catenin (92kDa)-2

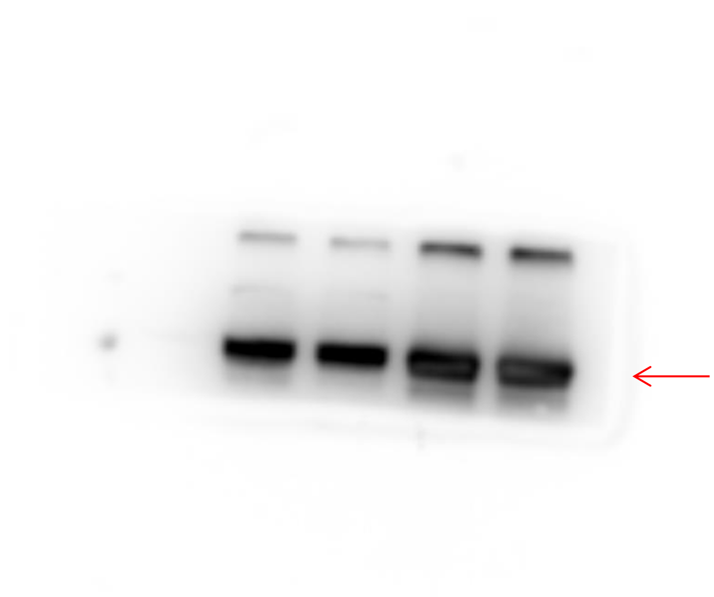

Figure 7.2 The Uncropped blot of β-catenin (92kDa)-2. The red arrow indicates the location of target bands.

The Uncropped blot of β-catenin (92kDa)-3

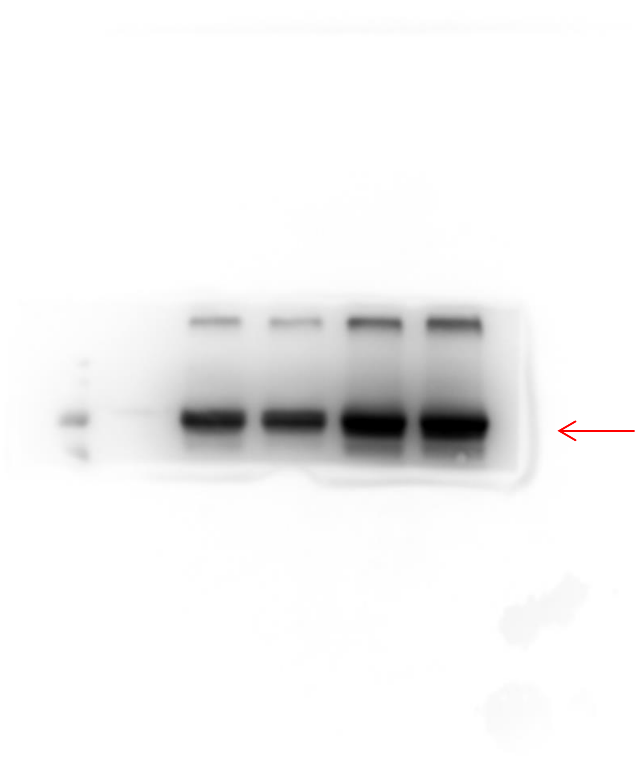

Figure 7.3 The Uncropped blot of β-catenin (92kDa)-3. The red arrow indicates the location of target bands.

The Uncropped blot of WNT7B (39kDa)-1

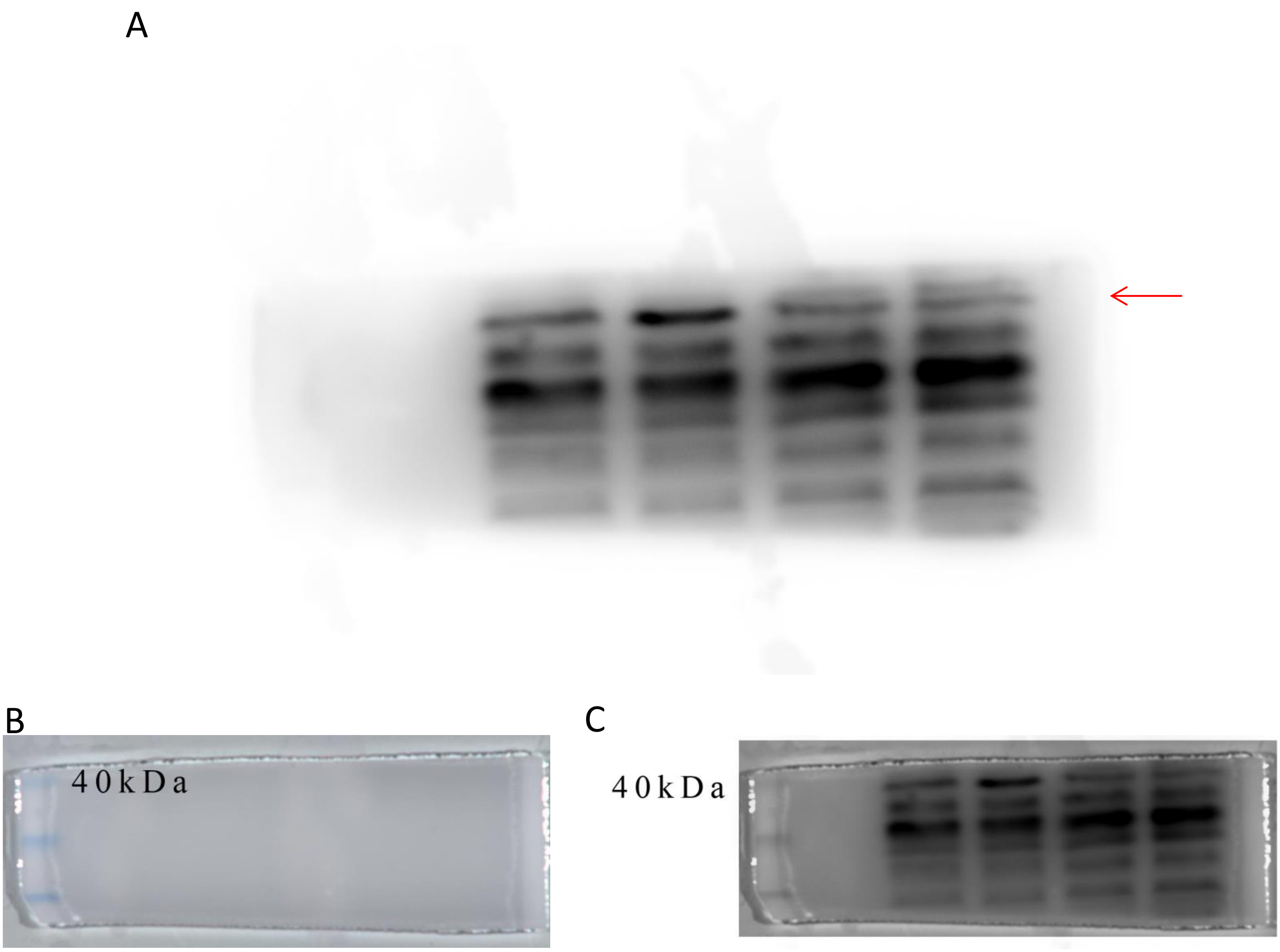

Figure 8.1 The Uncropped blot of WNT7B (39kDa)-1. (A) The Origin Picture of β-catenin. The red arrow indicates the location of target bands. (B) The Marker of β-catenin. (C) The Merge of Blot and Marker.

The Uncropped blot of WNT7B (39kDa)-2

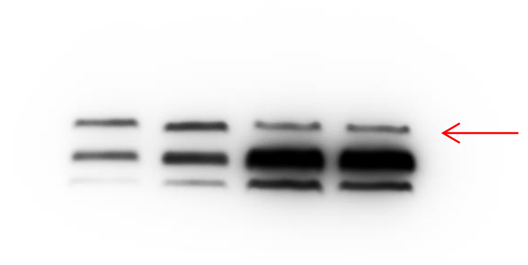

Figure 8.2 The Uncropped blot of WNT7B (39kDa)-2. The red arrow indicates the location of target bands.

The Uncropped blot of WNT7B (39kDa)-3

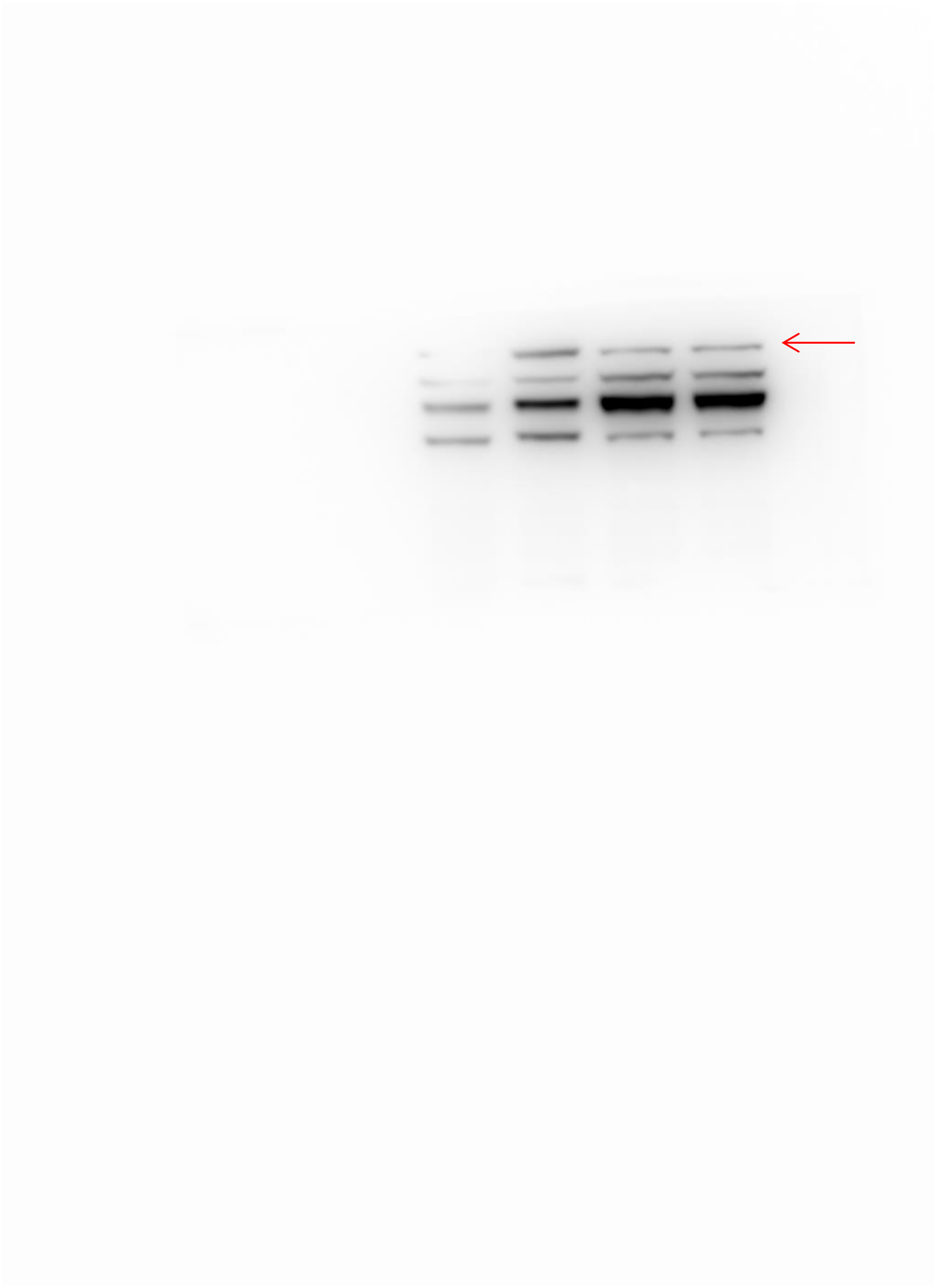

Figure 8.3 The Uncropped blot of WNT7B (39kDa)-3. The red arrow indicates the location of target bands.

The Uncropped blot of FZD8 (60kDa)-1

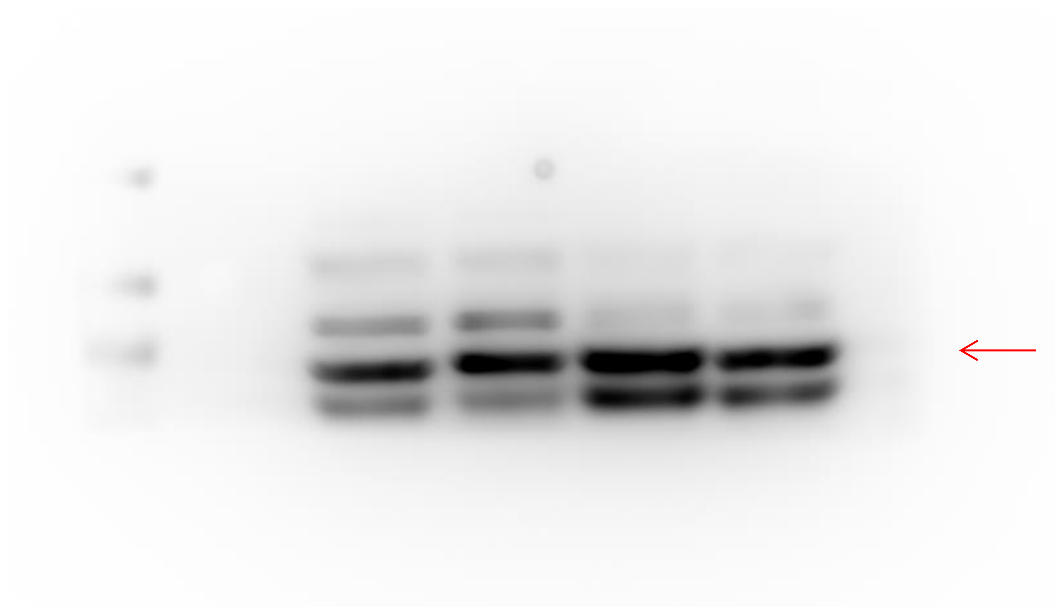

Figure 9.1 The Uncropped blot of FZD8 (60kDa)-1. The red arrow indicates the location of target bands.

The Uncropped blot of FZD8 (60kDa)-2

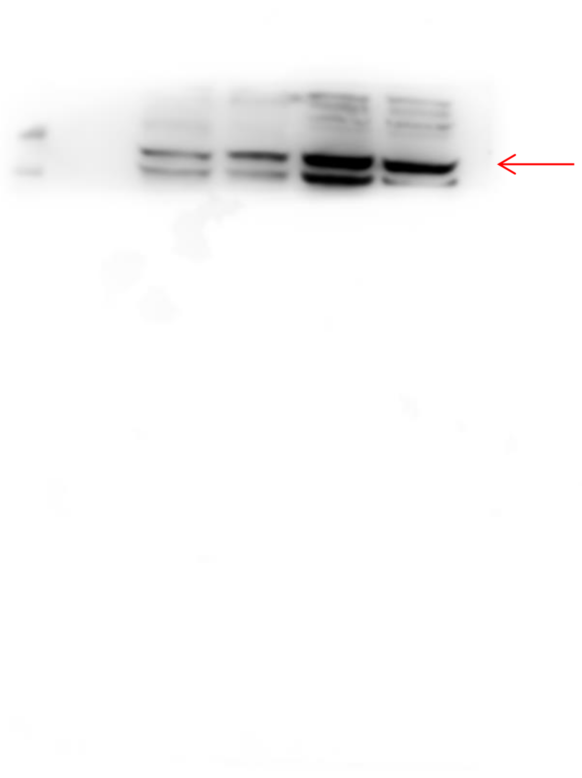

Figure 9.2 The Uncropped blot of FZD8 (60kDa)-2. The red arrow indicates the location of target bands.

The Uncropped blot of FZD8 (60kDa)-3

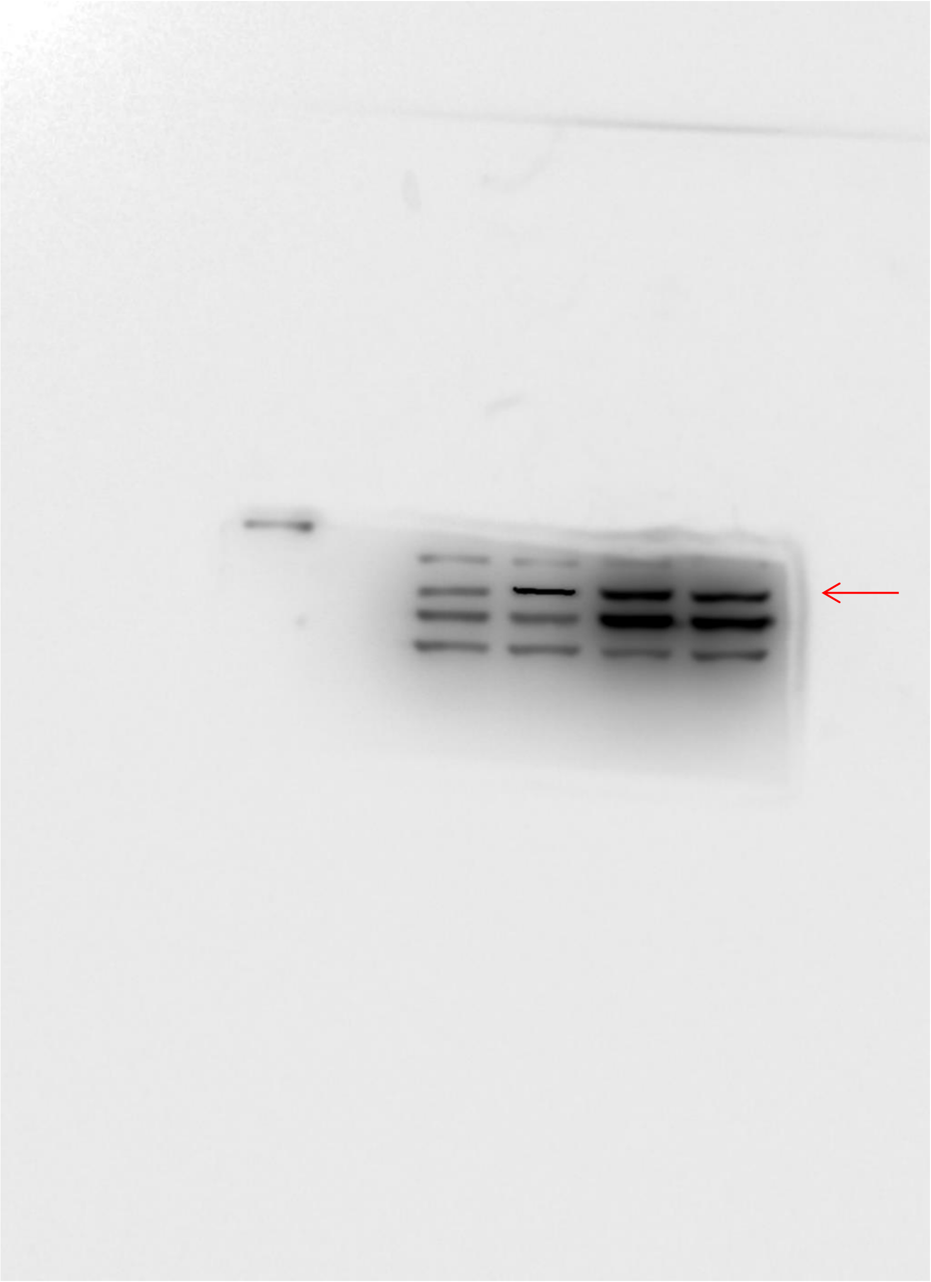

Figure 9.3 The Uncropped blot of FZD8 (60kDa)-3. The red arrow indicates the location of target bands.

The Uncropped Blot of β-actin (42kDa)

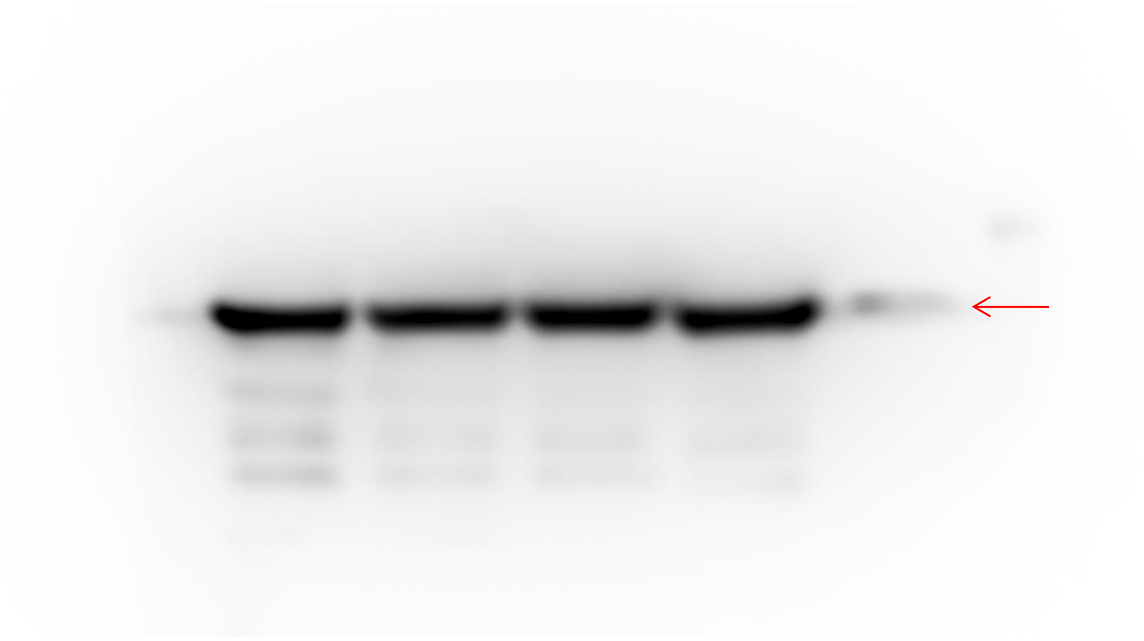

Figure 10 The Uncropped Blot of β-actin (42kDa). The red arrow indicates the location of target bands.

The Uncropped blot of Phospho-Caspase 3 (34kDa)-1

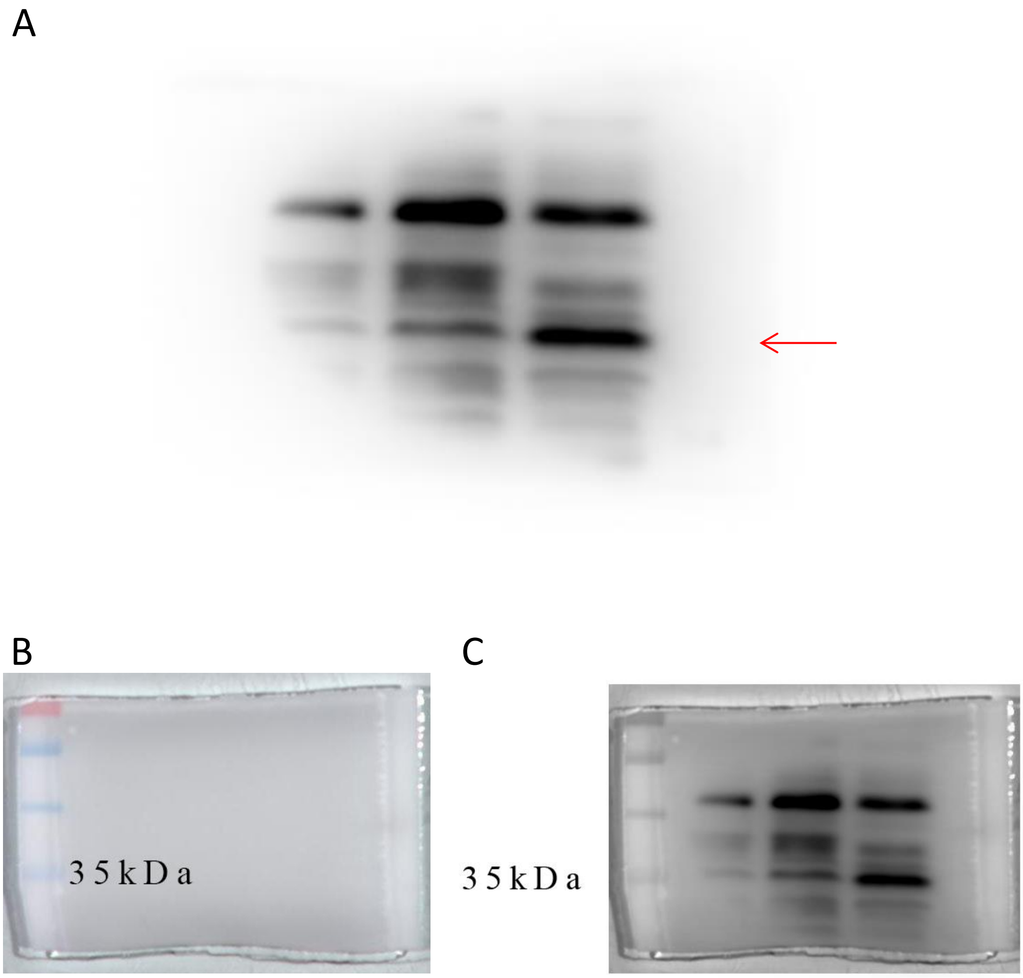

Figure 11.1 The Uncropped blot of Phospho-Caspase 3 (34kDa)-1. (A) The Origin Picture of Phospho-Caspase 3. The red arrow indicates the location of target bands. (B) The Marker of Phospho-Caspase 3. (C) The Merge of Blot and Marker.

The Uncropped blot of Phospho-Caspase 3 (34kDa)-2

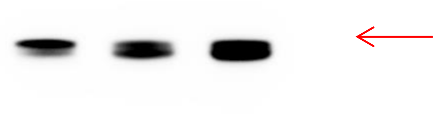

Figure 11.2 The Uncropped blot of Phospho-Caspase 3 (34kDa)-2.The red arrow indicates the location of target bands.

The Uncropped blot of Phospho-Caspase 3 (34kDa)-3

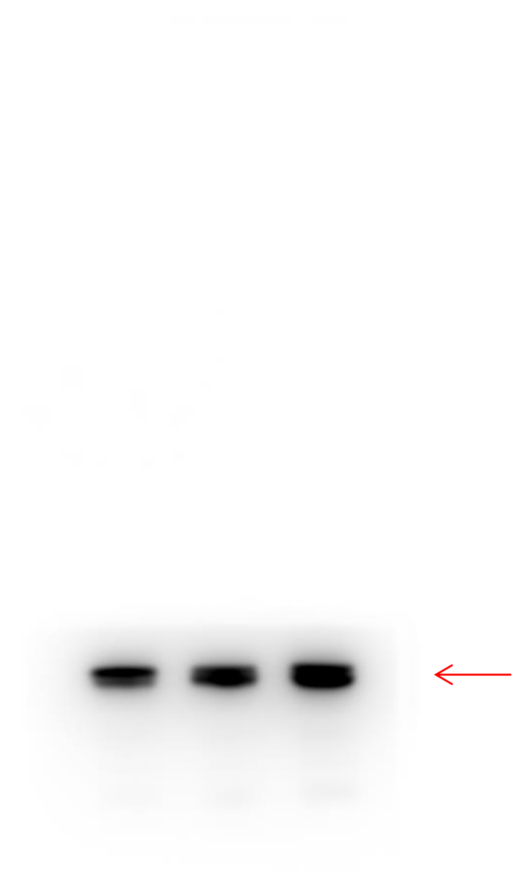

Figure 11.3 The Uncropped blot of Phospho-Caspase 3 (34kDa)-3.The red arrow indicates the location of target bands.

The Uncropped Blot of Caspase 3 (37kDa)-1

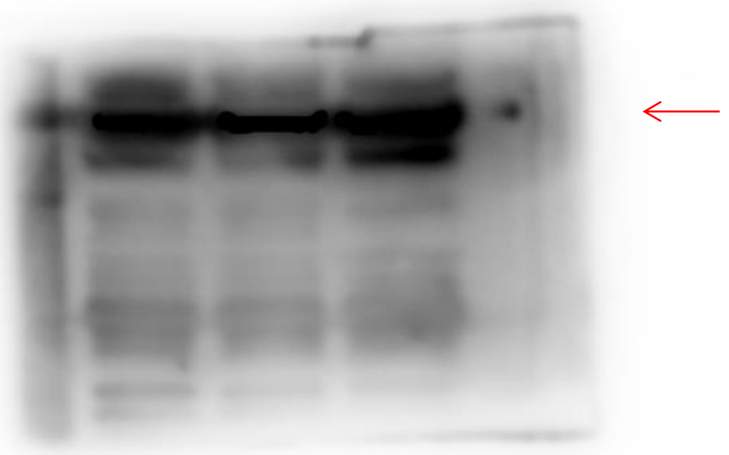

Figure 12.1 The Uncropped Blot of Caspase 3 (37kDa)-1. The red arrow indicates the location of target bands.

The Uncropped Blot of Caspase 3 (37kDa)-2

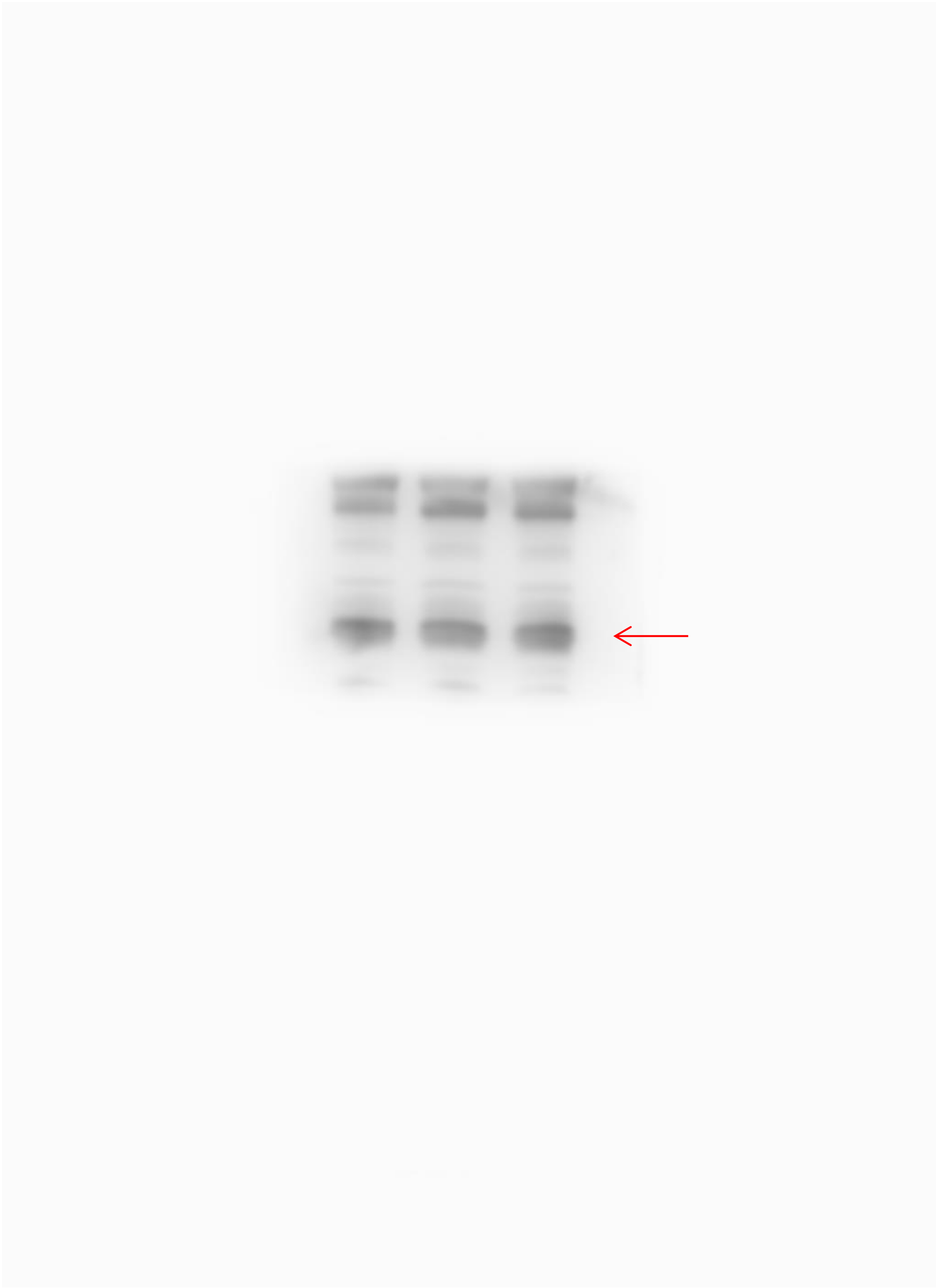

Figure 12.2 The Uncropped Blot of Caspase 3 (37kDa)-2. The red arrow indicates the location of target bands.

The Uncropped Blot of Caspase 3 (37kDa)-3

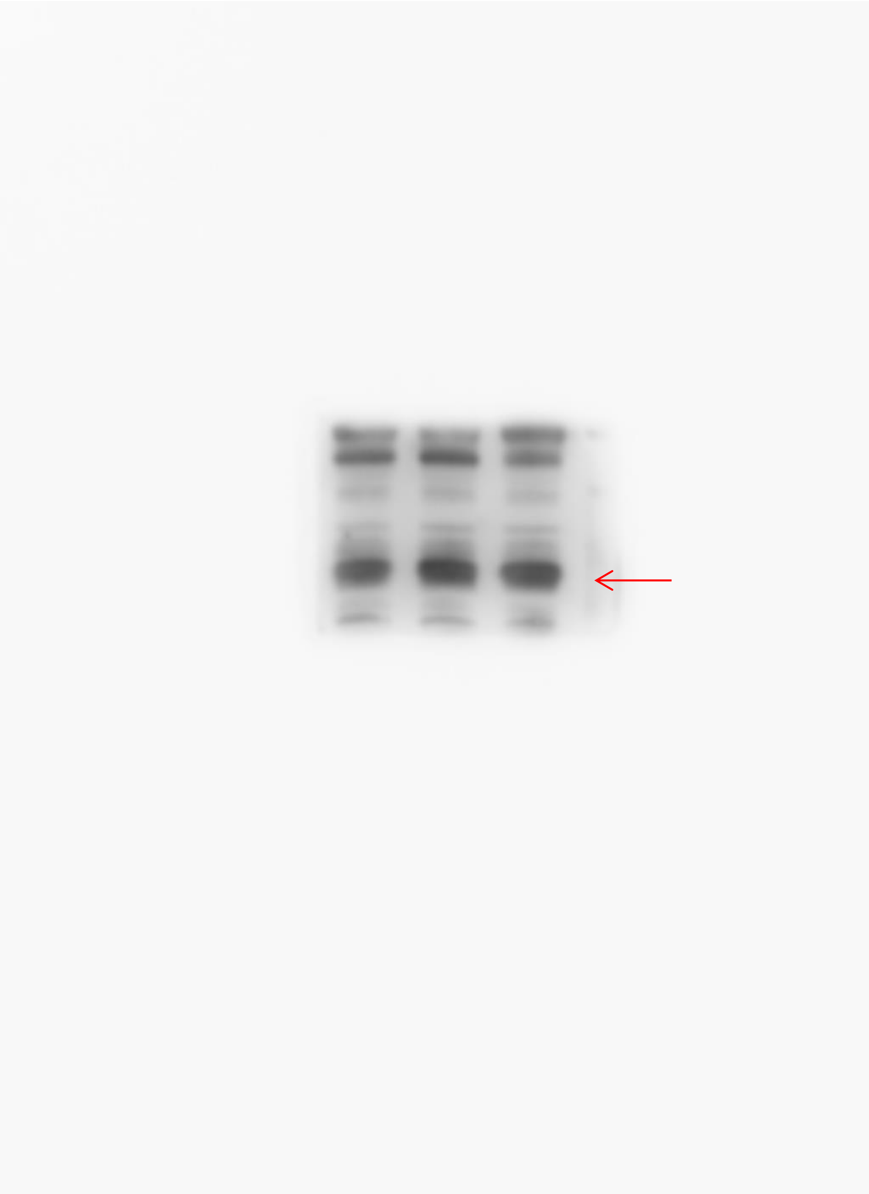

Figure 12.3 The Uncropped Blot of Caspase 3 (37kDa)-3. The red arrow indicates the location of target bands.

The Uncropped Blot of β-actin (42kDa)

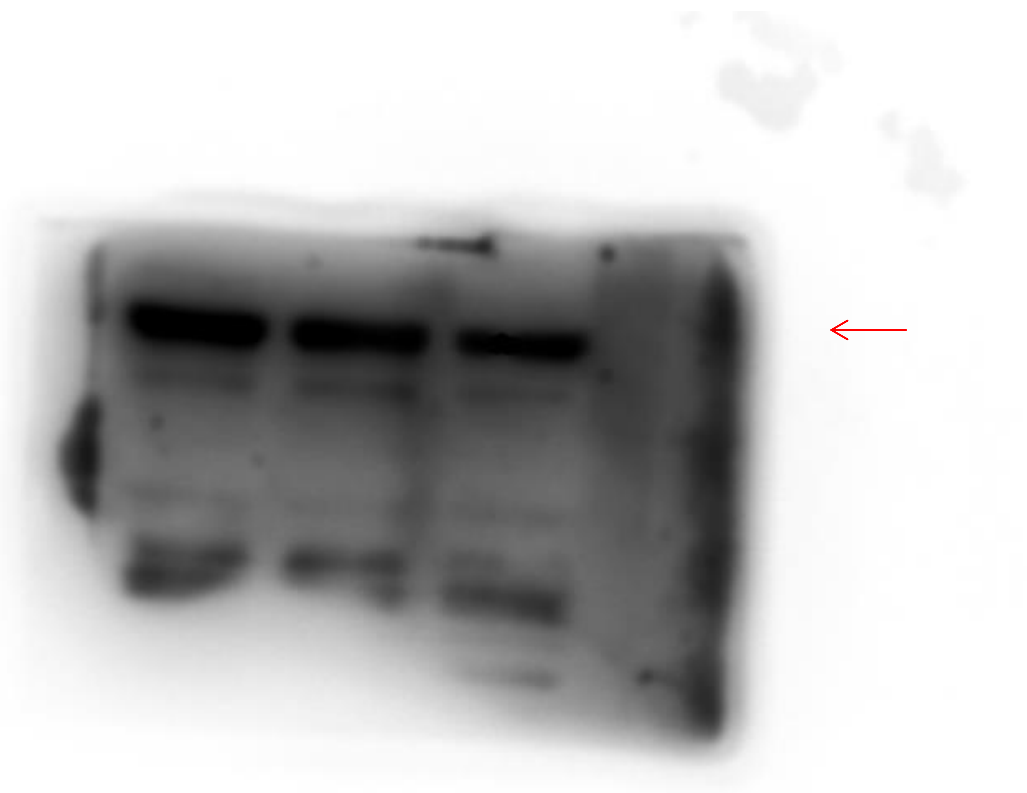

Figure 13 The Uncropped Blot of β-actin (42kDa). The red arrow indicates the location of target bands.
